# Role of nanomaterials for effective lignocellulosic biomass wastes conversion to hydrogen: a biorefinery perspective on commercialization and sustainability, challenges, and prospects

**DOI:** 10.3389/fmicb.2026.1793416

**Published:** 2026-04-07

**Authors:** Muhammad Usman, Faiqa Nadeem, Hina Ramzan, Nadeem Tahir

**Affiliations:** Collaborative Innovation Center of Biomass Energy, College of Mechanical and Electrical Engineering, Henan Agricultural University, Zhengzhou, China

**Keywords:** hydrogen, lignocellulosic biomass, nano-catalysts, photo fermentation, technology readiness level

## Abstract

The shift toward carbon-neutral energy systems has heightened the focus on sustainable hydrogen production from renewable resources, especially lignocellulosic biomass (LCB). This review comprehensively assesses the role of nanotechnology in photo-fermentative biohydrogen production, framed within an integrated biorefinery context. It highlights advancements in catalysis, underlying mechanisms, and the potential for commercialization. The inherent recalcitrance of lignocellulosic biomass, driven by the interplay of cellulose, hemicellulose, and lignin, necessitates effective pretreatment and hydrolysis to optimize the release of fermentable sugars. A specific emphasis is placed on the role of purple non-sulfur bacteria (PNSB) in photofermentation, highlighting how nitrogenase-mediated hydrogen production is augmented by nanophotocatalysts that enhance electron-transfer efficiency, enzyme activity, substrate conversion, and redox balance. Recent advances in nanomaterials, such as metal oxides, magnetic nanoparticles (MNPs), carbon-based nanostructures, and heterojunction photocatalysts, are examined in detail for their contributions to biomass depolymerization, metabolic regulation, and the optimization of light-harvesting processes. This review goes beyond mechanistic insights by incorporating assessments of Technology Readiness Level (TRL), techno-economic analysis (TEA), and life cycle assessment (LCA) to thoroughly evaluate scalability, environmental impact, and economic feasibility. A SWOT framework provides a clearer understanding of strengths, limitations, and barriers to commercialization, including catalyst deactivation, nanoparticle (NP) toxicity, constraints in reactor design, and the high costs associated with scale-up. While catalytic photo-fermentation remains in the early stages of development (TRL 1–3 for raw biomass systems), targeted advances in catalyst design, improvements in reactor efficiency, and the incorporation of circular bioeconomy principles could significantly enhance its potential for industrial application. This review outlines a detailed framework for enhancing nano-enabled photobiological hydrogen production to achieve sustainability, scalability, and economic competitiveness in biorefinery applications.

## Graphical Abstract

**Figure:**
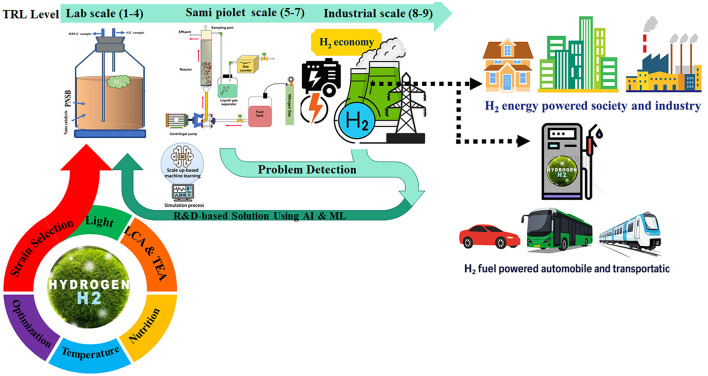

## 1 Introduction

Accelerated industrialization has led to a dramatic increase in energy demand, exacerbating environmental degradation and posing considerable obstacles to sustainable, long-term economic development (Raut et al., 2025a). Due to the overuse of petrochemicals and industrial processes, it is necessary to employ carbon-free, environmentally friendly alternative energy sources; however, CO_2_ emissions have increased to 1.83 trillion tons, resulting in ecological imbalance (Ahmed et al., 2020). The use of carbon-free, environmentally friendly alternative energy sources is unavoidable. Fossil fuels will continue to account for approximately 80% of the world's energy supply until 2040, despite the contributions of nuclear and renewable energy rising by 2.5% (Lin and Raza, 2020). These statistics present two challenges. The substitution of non-renewable resources that might become depleted within 100 years, environmental sustainability, and energy sustainability.

Consumption of fossil fuels produces substantial greenhouse gas (GHG) emissions, which significantly contribute to global warming and climate change. Between 2010 and 2021, global CO_2_ emissions increased from 33.4 billion metric tons to 36.4 billion metric tons, and this trend is expected to continue (Friedlingstein et al., 2022). Energy use and greenhouse gas (GHG) emissions are directly linked to climate change (Nadeem et al., 2023; Baiardi, 2020). The global economy will be substantially more significant than today by 2050, and energy consumption will increase by 80%. The global economy will be significantly larger than it is today by 2050, and energy consumption will increase by 80%. Energy consumption in the industrial sector and buildings, particularly in rapidly developing nations, accounts for three-quarters of the increase in energy consumption. The sustainability of a growing economy is threatened by environmental problems and rising energy prices (Li et al., 2021). Renewable energy sources are the focus of both climate change policy and economic development (Sher et al., 2021; Olabi and Abdelkareem, 2022) sources, including solar and wind, is widely acknowledged to mitigate the harmful effects of climate change (Eitan, 2021) and economic growth (Kumar, 2020).

The importance of energy is also emphasized in the United Nations Sustainable Development Goals (UND ‘G). Goals 7 (Affordable and Clean Energy) and 13 (Climate Action) both underscore the significance of new and renewable energy in driving global transformation (Aziz et al., 2021; Usman et al., 2024). Biomass is a viable option because it is readily available, renewable, does not contribute to carbon emissions, and supports environmental sustainability globally (Chen et al., 2021). Food is currently the primary end-use of biomass, followed by animal feed, chemicals, electricity, and transportation fuels. This represents 13% of global eventual energy consumption, accounting for an additional 5% of the overall eventual energy consumption (Popp et al., 2021). Biohydrogen, a promising renewable energy source, is abundant, pollution-free, and low-cost. It produces no carbon dioxide (CO_2_) emissions and achieves maximal energy density until hydrogen (H_2_) combustion produces only H_2_O vapor (Rasmey et al., 2026; Tahir et al., 2024).

The growing global energy demand has prompted the extensive exploration of alternative energy sources. Among these options, biomass stands out as a promising and renewable resource for energy production, positioning it as the fourth-largest energy source after coal, oil, and natural gas (Wu et al., 2012). Biomass encompasses a range of biologically derived materials and sources, including forestry, agriculture, and waste products. Its abundance and versatility make it an attractive choice for meeting energy requirements (Raut et al., 2025a; Usman et al., 2026). Lignocellulosic biomass (LCB) refers to a type of biomass that is characterized by its composition of lignin, hemicellulose, and cellulose (Raut and Mishra, 2025). They can be classified into two main categories: woody and non-woody. Woody biomass can be further divided into hardwoods and softwoods, distinguished by reproductive characteristics: hardwoods are angiosperms and softwoods are gymnosperms (Wu et al., 2012). Examples of hardwoods include beech, mahogany, maple, and teak, whereas softwoods include cedar, pine, juniper, and spruce. Non-woody biomass comprises agricultural residues, the Poaceae or Gramineae family grasses, and non-woody fibers, such as cotton (Hitam and Jalil, 2020).

Energy efficiency, financial viability, and minimal environmental impact are hallmarks of bioprocessed LCB and are essential for enhancing the energy efficiency of biomass (Shahzaib et al., 2024). By 2050, the transition to a sustainable global energy system will rely heavily on the H_2_ energy sector sustainable energy system (Staffell et al., 2019; Zulfiqar et al., 2026). A wide range of organisms can produce biological hydrogen in diverse environments, including cyanobacteria, photo fermentation (Raut and Mishra, 2024), photosynthetic bacteria, hydrogen-producing bacteria, and green microalgae (Xia et al., 2019). Hydrogen can be produced from organic matter, such as biomass or waste. Photobiological hydrogen production and dark fermentation are the two most common biomass conversion methods (Yukesh Kannah et al., 2021; Usman et al., 2025). The energetic value of hydrogen, the amount of energy produced from 1 kg of material, is the highest of any known fuel save those formed by nuclear fission. Hydrogen, on the other hand, is an environmentally friendly fuel because it produces no CO_2_ when burned (Kucharska et al., 2018). Therefore, bridging the gap between laboratory innovation and industrial feasibility requires a systematic evaluation of catalyst design, reactor engineering, sustainability metrics, and commercialization pathways.

Economic viability and environmental sustainability are essential for assessing the commercialization potential of biohydrogen technologies, as they help identify significant opportunities and obstacles associated with large-scale deployment. Numerous studies and reviews have examined various facets of biohydrogen production, including process optimization, economic viability, and system integration strategies. This article critically analyzes the impact of the development of nanomaterials and improved optimization techniques on biohydrogen generation, particularly in light of recent advances in nanotechnology. Additionally, a Strengths, Weaknesses, Opportunities, and Threats (SWOT) analysis is included to provide a strategic perspective on biological biohydrogen generation, particularly with respect to natural resource use and system sustainability. This review integrates Technology Readiness Levels (TRL), Life Cycle Assessment (LCA), and Techno-Economic Analysis (TEA) frameworks to thoroughly evaluate technological maturity and development status. This comprehensive assessment elucidates the current state of biohydrogen technologies and identifies significant research gaps, emerging trajectories, and avenues for technological progress toward sustainable commercialization.

## 2 The chemistry of lignocellulosic biomass

The world's most abundant bioresource is LCB, which produces 1.3 billion tons annually (Baruah et al., 2018). Biomass is abundant in cellulose, making it a sustainable substrate for hydrogen synthesis via natural routes (Al-Battashi et al., 2019). Cellulose, hemicellulose, and lignin are the main components of lignocellulosic biomass, and their availability in free form is essential for bioconversion (Akubude et al., 2021; Sarangi and Nanda, 2020). The richest renewable resource, lignocellulosic waste biomass, is used to make byproducts (Akbarian et al., 2022; Hu et al., 2017; Patinvoh et al., 2017). Lignocellulosic biomass, specifically hemicellulose and cellulose structures (also known as holocellulose), consists of sugar monomers that microorganisms can utilize. These sugars can undergo biological pathways to convert them into diverse biofuels. Cellulose, a primary component of lignocellulosic biomass, is a homopolymer composed solely of glucose monomers (Brodeur et al., 2011). β-(1 → 4) glycosidic linkages connect glucose molecules within cellulose. The cellulose chain is often referred to as β*-1,4-*glucan. These chains are tightly packed into minute, highly elongated structures called microfibrils. These microfibrils further aggregate into lattices, creating regions of cellulose fibers that are primarily inaccessible to enzymes (Kumar et al., 2008).

Hemicellulose is another essential component of lignocellulosic biomass, containing sugars that microorganisms can utilize. It is a heteropolymer composed of various sugars, including pentoses (xylose and arabinose), hexoses (mannose, glucose, and galactose), and uronic acids (4-O-methyl glucuronic acid and galacturonic acid). Hemicellulose heterogeneity results in diverse structures across biomass types (Okolie et al., 2021). Hardwood predominantly contains glucuronoxylan, which is the major hemicellulose. The glucuronoxylan backbone consists of xylose units linked by β-(1 → 4) glycosidic linkage. Additionally, acetylation occurred at the C2 and C3 positions of the xylose molecules. α-(1 → 2) linkage connected the side chains of 4-O-methylglucuronic acid attached to the xylose backbone. On the other hand, softwoods primarily contain galactoglucan as the main hemicellulose. As the name suggests, the galactoglucan backbone comprises mannose and glucose units with galactose and acetyl groups serving as side chains. Grasses, including cereals, consist mainly of glucuronoarabinoxylan as their major hemicellulose, with xylose as the backbone (Hassan et al., 2018).

Lignin is an essential component of LCB and is unique as it is not composed of sugars. It is the second most abundant biopolymer after cellulose. Lignin is an amorphous polymer with a diverse structure that depends on the type of biomass and environmental conditions. Its main constituents are three phenylpropane units, p-hydroxyphenyl (H), guaiacol (G), and syringyl (S), which are derived from aromatic alcohols, namely p-coumaric, coniferyl, and sinapyl alcohols (Cai et al., 2017; Ramzan et al., 2023). Although the sugar monomers present in the holocellulose fraction of biomass are of interest for biofuel production via biological pathways, extracting them remains challenging. All three components of lignocellulose are intricately intertwined and are resistant to hydrolysis. Cellulose is highly crystalline, and its microfibrils are tightly interconnected with hemicellulose. Moreover, lignin fills structural gaps, provides additional strength, increases wall hydrophobicity, and acts as a barrier to hydrolytic enzymes (Acharjee et al., 2009).

**Figure 1:**
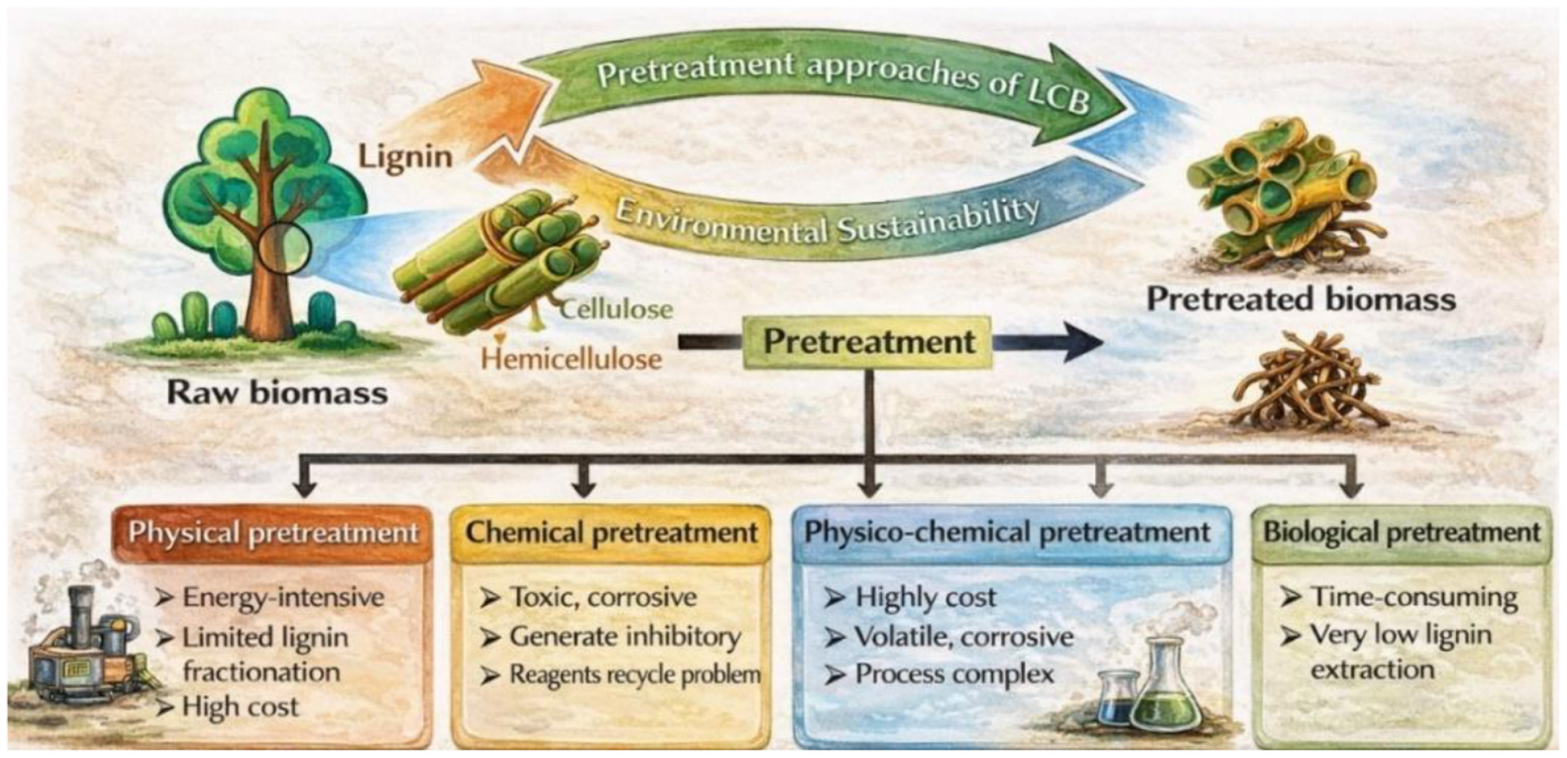
Classification of physical, chemical, physico-chemical, and biological pretreatment techniques for lignocellulosic biomass, with their limitations and effects on lignin removal and biomass structure.

In recent years, considerable focus has been placed on pretreatment procedures to improve the efficiency of subsequent hydrolysis, thereby optimizing the release of fermentable sugars from the cellulose and hemicellulose components of lignocellulosic biomass. Delignification is crucial in this context, as it dismantles the structural barrier created by lignin, enhances cellulose accessibility, and augments enzymatic digestibility, an indispensable phase in biochemical conversion processes (Thanigaivel et al., 2022). Delignification is a crucial phase in the processing of lignocellulosic biomass, as it dismantles the structural barrier created by lignin, hence increasing cellulose accessibility and enhancing sensitivity to enzymatic hydrolysis, a vital stage in biochemical conversion pathways. The appropriate pretreatment approach depends on the specific composition of the feedstock, particularly the relative amounts of cellulose, hemicellulose, and lignin, as well as considerations of economic viability and environmental sustainability, as explained in Tables 1, 2. To deem biofuel production sustainable, it is imperative to address key issues, including reducing chemical inputs, intensifying processes, and minimizing power, water, and overall energy consumption. Figure 1 illustrates the pretreatment methods for lignocellulosic biomass, which are classified as physical, chemical, physicochemical, and biological processes, along with their principal limitations and effects on biomass structure and lignin removal (Raut and Mishra, 2025; Pandey et al., 2026).

**Table 1:** Comparative overview of different pre-treatment methods of delignification of biomass for biohydrogen production.

Pretreatment category	Common methods	Key advantages	Major limitations	References
Physical	Milling, grinding, extrusion, microwave (MW), steam explosion (SE), hot water, ultrasonication	•Rapid hydrolysis rate •Increased surface area •Improved substrate accessibility •Some methods highly effective (e.g., MW, hot water)	•Very high energy demand •Expensive equipment •Potential formation of inhibitory compounds (phenolics, furans) •Economically less viable at large scale	(Sitotaw et al., 2023; Júnior et al., 2025; Fan et al., 2025a; Silva et al., 2025)
Chemical	Acid (H_2_SO_4_), alkali (NaOH, Ca(OH)_2_), alkaline H_2_O_2_, ammonia, organosolv	•Highly effective delignification •Fast reaction rates •Significant BMP enhancement (up to 100%) •Improved solubilization of hemicellulose/lignin	•High chemical cost •Disposal challenges •Formation of inhibitory compounds •High energy input (temperature dependent)	(Martins et al., 2026; Fan et al., 2025b; Bedru et al., 2025; Wang et al., 2025; Shahzaib et al., 2025)
Biological	Fungal (e.g., *Pleurotus, Trichoderma*), bacterial, enzymatic, and microaerobic pretreatment	•Low energy consumption •Eco-friendly •Minimal inhibitor formation •Cost-effective under mild conditions	•Slow hydrolysis rate (10–14 days) •Less effective as a standalone •Variability in performance •Enzyme cost (in some cases)	(Salhotra et al., 2026; Kumar et al., 2025; Lyu et al., 2025; Li et al., 2025a)
Combined/ Physicochemical	Steam explosion + alkali, thermo-alkaline, extrusion + chemical, hydrothermal	•Fast and effective •Enhanced methane yields (>200% in some cases) •Balanced performance	•Process complexity •Moderate to high energy demand •Possible inhibitor generation •Auxiliary equipment cost	(Shahzaib et al., 2025; Gladysheva, 2025; Wozniak et al., 2025)

**Table 2:** Aggregated comparison of the pretreatment methods (Singh et al., 2015).

Method	Hydrolysis rate	Energy use	Effectiveness	Cost	Toxic by-products
Chemical	Fast	High	Very effective	Very expensive	Yes
Biological	Slow	Very low	Less effective	Cost-effective	No
Physical	Very fast	Very high	Moderately effective	Very expensive	Yes
Combined	Fast	Moderate	Effective	Cost-effective	Yes

Lignocellulose is plant biomass composed of cellulose and hemicellulose polysaccharides bonded to lignin, a heteropolymer containing phenylpropane monomers (Sołowski et al., 2020). The common substrates in lignocellulose feedstock that occur in the majority are monosaccharides glucose (Equation 1), xylose (Equation 2), and arabinose (Equation 3). Bioconversion of these to biohydrogen (in the absence of molecular nitrogen) occurs through the following chemical reactions:


$$\begin{array}{l} C_{6}H_{12}O_{6}+\;H_{2}O+Light\;\to \;12H_{2\;}+\;6CO_{2\;}895.8\;kJ/mol \end{array}$$ (1)

$$\begin{array}{l} C_{5}H_{10}O_{5}+\;5H_{2}O+\;light\;\to \;10H_{2\;}+\;5CO_{2}\;746.0\;kJ/mol \end{array}$$ (2)

$$\begin{array}{l} C_{5}H_{10}O_{5}+\;5H_{2}O+\;light\;\to \;5H_{2\;}+\;5CO_{2}\;746.0\;kJ/mol \end{array}$$ (3)

Complete oxidation of glucose (Equation 1) yields more hydrogen than pentose sugars, xylose, and arabinose (Equations 2, 3). The hexose substrate also has greater potential free energy than pentose sugars, which can be captured via biochemical pathways and utilized for biohydrogen production (Mabutyana and Pott, 2021). Purple non-sulfur photosynthetic bacteria (PNSB), such as Rhodobacter species, are used to convert glucose or organic acids to H_2_ and CO_2_ under anaerobic conditions, as shown in Equation 1. Theoretical H_2_ yields from butyric acid, propionic acid, malic acid, succinic acid, lactic acid, and acetic acid in photo fermentation pathways are given in Equations 4–9.


$$\begin{array}{l} CH_{3}CH_{2}CH_{2}COOH\;+\;6H_{2}O\;\to {10H}_{2\;}+\;{4CO}_{2} \end{array}$$ (4)

$$\begin{array}{l} CH_{3}CH_{2}COOH\;+\;4H_{2}O\;\to \;7H_{2}\;+\;3CO_{2} \end{array}$$ (5)

$$\begin{array}{l} COOHCH_{2}CHOHCOOH+{3H}_{2}O\;\to \;{6H}_{2}+\;{4CO}_{2} \end{array}$$ (6)

$$\begin{array}{l} COOHCH_{2}CH_{2}COOH\;+{4H}_{2}O\to \;{7H}_{2}+{4CO}_{2} \end{array}$$ (7)

$$\begin{array}{l} CH_{3}CHOCOOH\;+\;3H_{2}O\;\to {6H}_{2\;}+\;{3CO}_{2} \end{array}$$ (8)

$$\begin{array}{l} CH_{3}COOH\;+\;2H_{2}O\;\to {4H}_{2\;}+\;{2CO}_{2} \end{array}$$ (9)

These bacteria capture light to convert organic acids to H_2_ using oxygen-sensitive nitrogenase. The production of H_2_ is limited by low nitrogenase activity and suppression of their expression in the presence of NH_4_. The photo fermentation process is conducted under light illumination. Low light conversion efficiency, C/N ratio, heavy metal ions, oxygen inhibition of nitrogenase, and toxicity to PNSB caused by high substrate concentrations are limiting factors for achieving optimal H_2_ yields (Bhatia et al., 2021; Mishra et al., 2019). Following dark fermentation, photofermentation, aided by the photosynthetic purple non-sulfur (PNS) bacteria, is another prominent and widely used method for biological hydrogen generation (Reungsang et al., 2018). Found that fatty acids, which are volatile, short, and long, may be effectively transformed into hydrogen and associated metabolites by the nitrogenases in the presence of sunlight by these bacteria (Pandey et al., 2021). Furthermore, such bacteria can use a broad range of biomass substrates to produce H_2_ effectively via light-driven processes. All *Rhodobacter* species, *Rhodobacter capsulatus, Rhodovulums ulfidophilum* W1S, *Rhodopseudomonas palustris*, and *Rhodobacter sphaeroides* are recognized for producing H_2_ via light fermentation with high yields, utilizing the substrate VFAs (Usman et al., 2026). Substrate and microorganisms are critical for a high production of H_2_ in light fermentation (Assawamongkholsiri et al., 2019). The majority of cell growth and H_2_ generation by light-fermentative bacteria occur at temperatures between 30 and 40 °C, pH between 5.0 and 7.0, and normal atmospheric pressure, using various substrates and light sources with wavelengths of 850, 805, and 522 nm (Pandey et al., 2021). In photo-fermentation, optimizing physicochemical parameters is as important as optimizing the substrate and microorganisms, resulting in high H_2_ yields (Soto et al., 2019). The majority of cell growth of light fermentative bacteria and H_2_ generation occurs at temperatures between 30 and 40 °C, pH between 5.0 and 7.0, and normal air pressure, using various substrates and light sources with wavelengths of 850, 805, and 522 nm (Liu et al., 2015). Moreover, because no pH adjustment is necessary, the dark fermentation effluent may be used directly for light fermentation in the next step or in a sequential procedure. In photo-fermentation, modification of physicochemical parameters, in addition to substrate and microorganisms, is crucial for high H_2_ generation (Soto et al., 2019).

PNSB prefer photoheterotrophic growth, relying on an organic carbon source, particularly small organic acids. Hydrogen production occurs specifically during photoheterotrophic growth, although PNSB can adapt to different growth modes, such as photoautotrophic, respiratory, fermentative, or chemotrophic conditions, depending on factors including light availability, carbon source type, and oxygen availability (Sivaramakrishnan et al., 2021). Non-oxygenic photobiological hydrogen production is performed by PNSB. These bacteria can produce H_2_ during their growth in the presence of organic carbon sources, utilizing energy from sunlight. The electrons generated during substrate oxidation are converted into H_2_ by the enzyme nitrogenase. Unlike in oxygenic photosynthesis, PNSB do not produce oxygen (O_2_) as a byproduct, so the repression of nitrogenase by O_2_ is not a concern in their photo fermentative hydrogen production. The process of H_2_ evolution in PNSB during photoheterotrophic growth is well-studied. To regulate the H_2_ cycling within the bacterial cells, PNSB, like cyanobacteria, contain uptake and bidirectional hydrogenases (Rathi et al., 2022). Biohydrogen production through photo fermentation in purple non-sulfur bacteria (PNSB) using lignocellulosic materials encompasses various molecular mechanisms. The following is a general overview of the molecular process.

**Figure 2:**
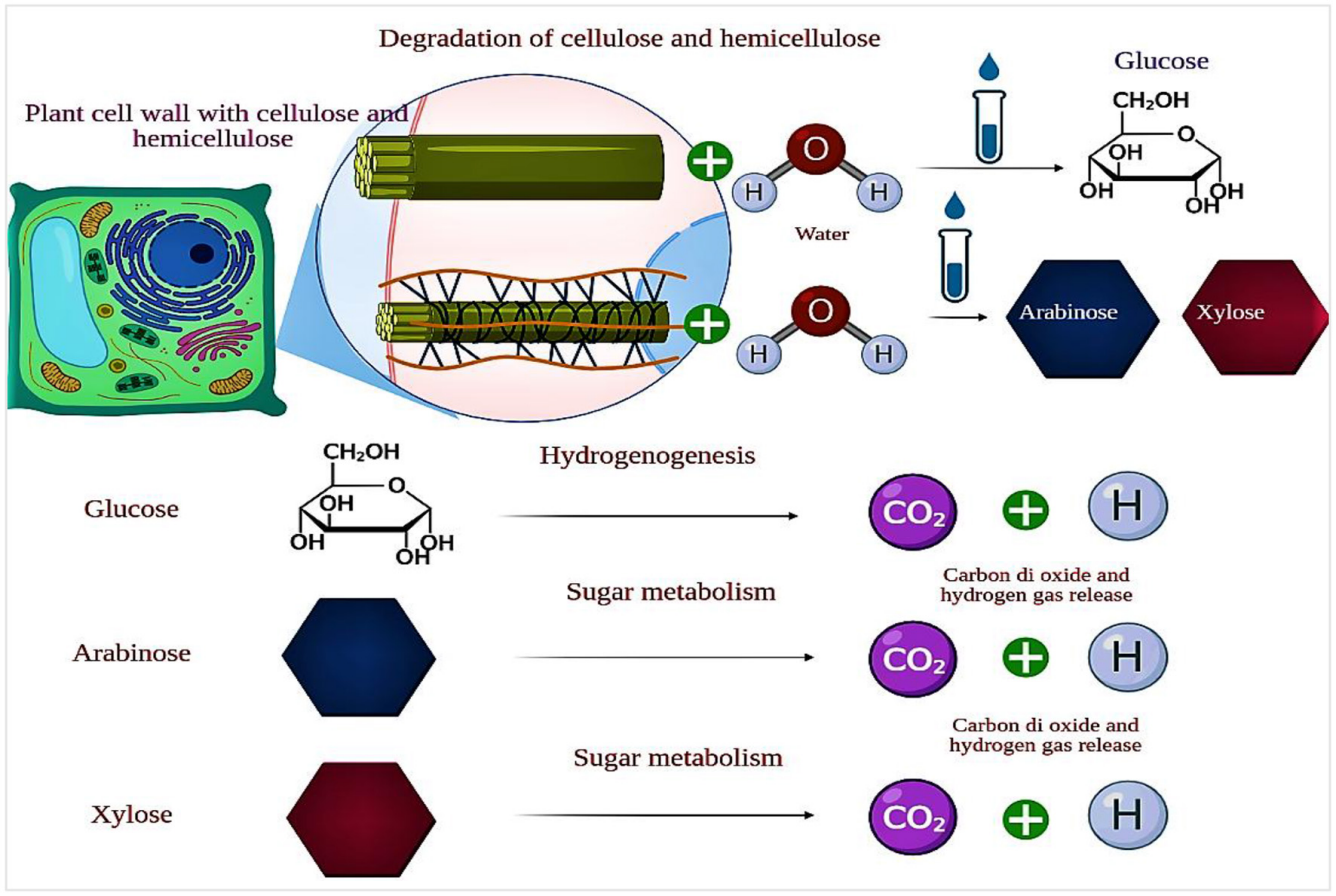
Conversion of cellulose and hemicellulose into simple sugars and hydrgengenesis of simple sugars into hydrogen gas and CO2.

Hydrolyzed cellulose sugars, also known as organic acids, primarily glucose, function as the principal substrate for purple non-sulfur bacteria during photo fermentation (Figure 2). These bacteria can undergo anaerobic growth and utilize organic compounds as electron donors via light-initiated electron transport into their photosystems (Wu et al., 2012; Putatunda, 2022). PNSB possess specialized pigments, like bacteriochlorophylls, that enable them to capture light energy. Through photosynthesis, light energy is converted into ATP, which serves as the energy source for various cellular functions. The photosynthetic apparatus in PNSB is localized within the intracytoplasmic membranes, which are invaginations of the cytoplasmic membrane that form parallel lamellae beneath it. This apparatus comprises a photosystem, a series of electron-transport proteins (including cytoplasmic cytochrome c, lipid-soluble quinones, and the cytochrome b/C1 complex), and a transmembrane ATP synthase. Within the photosystem are light-harvesting complex 1, light-harvesting complex 2, and a reaction center. These complexes capture light in the visible (450–590 nm) and near-infrared (800–875 nm) ranges, transferring the excitation energy to the reaction center, initiating cyclic electron transfer (Ghosh et al., 2017).

These complexes contain different types of carotenoids and bacteriochlorophyll a, forming protein-pigment complexes. The biosynthesis of the photosynthetic apparatus is primarily regulated by oxygen (O_2_) and light. During aerobic growth, bacteriochlorophyll synthesis is suppressed. Once the oxygen tension is eliminated, the synthesis resumes. Light intensity and quality also play a role in controlling the biosynthesis of the photosynthetic apparatus. Under low light intensity, photosystem biosynthesis increases to capture more light energy, while at high light intensity, less photosystem is synthesized. The photosystem in PNSB lacks the capacity to split water, resulting in the absence of oxygen evolution and making it particularly suitable for biohydrogen production. Electrons released during the oxidation of organic carbon pass through a series of electron carriers, thereby pumping protons across the membrane (Sagir et al., 2017). This creates a proton gradient, which drives ATP production by ATP synthase. The electrons are either used to replenish the quinone pool or donated to Ferredoxin, which transfers them to the nitrogenase enzyme for the reduction of molecular nitrogen to ammonia. In the absence of molecular nitrogen, nitrogenase functions as a hydrogenase, catalyzing proton reduction using electrons derived from ferrodoxin. This enables the storage of electrons from organic compounds as H_2_ via light energy (Wu et al., 2012).

The biohydrogen production process in PNSB is primarily mediated by nitrogenase. Nitrogenase catalyzes the reduction of nitrogen gas (N_2_) to ammonia (NH_3_) and also possesses inherent hydrogenase activity, producing H_2_ as a byproduct, as illustrated in Equation 10. The reaction can be summarized as follows:


$$\begin{array}{l} N_{2\;}+8\;H\;+8e^{-}+16\;ATP\;\to \;NH_{3}+H_{2}+16\;ADP+16P_{i\;} \end{array}$$ (10)

However, when nitrogen is limited, the nitrogenase enzyme functions as a hydrogenase and catalyzes the reduction of protons (*H*^+^) to form molecular hydrogen. This reaction consumes 2*H*^+^, 2*e*^−^, and 4 ATP, and can be represented as Equation 11:


$$\begin{array}{l} {2H}^{+}+2\;e^{-}+4\;ATP\;\to H_{2}\;+4\;ADP+4P_{i} \end{array}$$ (11)

Therefore, under nitrogen-limiting conditions, with the same energy requirement, four times as much hydrogen can be produced as nitrogen. PNSB also possesses a membrane-bound [Ni-Fe]-hydrogenase that is involved in the uptake and oxidation of H_2_, as shown in Equation 12. The reaction catalyzed by this enzyme is reversible:


$$\begin{array}{l} H_{2}\;\to {2H}^{+}+2\;e^{-} \end{array}$$ (12)

To produce H_2_ gas, the ATP flux to the cell must exceed the amount of ATP required for growth. Bacteria generate hydrogen when there is an excess of reducing power to maintain cellular redox balance. Three main metabolic pathways compete for electrons: CO_2_ fixation, N_2_ fixation/H_2_ production, and polyhydroxybutyrate (PHB) biosynthesis. In purple non-sulfur bacteria, CO_2_ serves as an electron sink under photoheterotrophic conditions to eliminate excess reducing equivalents and maintain redox homeostasis. The Calvin-Benson-Bassham (CBB) pathway is employed by PNSB to fix CO_2_, consuming ATP and NADH in the process. While the primary function of the CBB pathway is to provide carbon for the cell during photoautotrophic growth on CO_2_, it primarily serves redox-balancing purposes during photoheterotrophic growth on organic carbon. The key regulatory enzyme in CO_2_ fixation is ribulose-1,5-bisphosphate carboxylase/oxygenase (RuBisCO), which facilitates the conversion of RuBP (ribulose-1,5-bisphosphate) into glyceraldehyde-3-phosphate (Ghosh et al., 2017).

Several genes are involved in the process of photo fermentation in PNSB. Here are some of the key genes associated with photo fermentation in PNSB: *puf Operon*: the *puf operon* includes genes encoding the components of the photosynthetic apparatus, such as the light-harvesting complexes (LH1 and LH2), reaction center proteins, and associated pigment-binding proteins. *nif Operon*: the *nif operon* consists of genes responsible for nitrogen fixation in PNSB. These genes encode nitrogenase enzymes, which catalyze the reduction of nitrogen gas (N_2_) to ammonia (NH_3_). The nitrogenase enzyme is essential for the production of H_2_ gas during photo fermentation. hox Operon: the *hox operon* contains genes that encode hydrogenase enzymes in PNSB. These enzymes are involved in the production and utilization of hydrogen gas. They catalyze the reversible conversion of protons (H^+^) to molecular H_2_ and vice versa. *cbb Operon*: the *cbb operon* includes genes associated with CO_2_ fixation. These genes encode enzymes involved in the Calvin–Benson–Bassham (CBB) pathway, which is responsible for incorporating CO_2_ into organic compounds during photo fermentation, *fdx Operon*: the *fdx operon* comprises genes encoding ferredoxin proteins. Ferredoxins play a crucial role in electron transfer reactions and are involved in transferring electrons from various metabolic pathways, including photosynthesis and hydrogen production. It's important to note that the specific genes involved in photofermentation can vary among different species and strains of PNSB. The presence and organization of these genes in the genome contribute to the metabolic capabilities of PNSB for hydrogen production and CO_2_ fixation during photofermentation.

**Figure 3:**
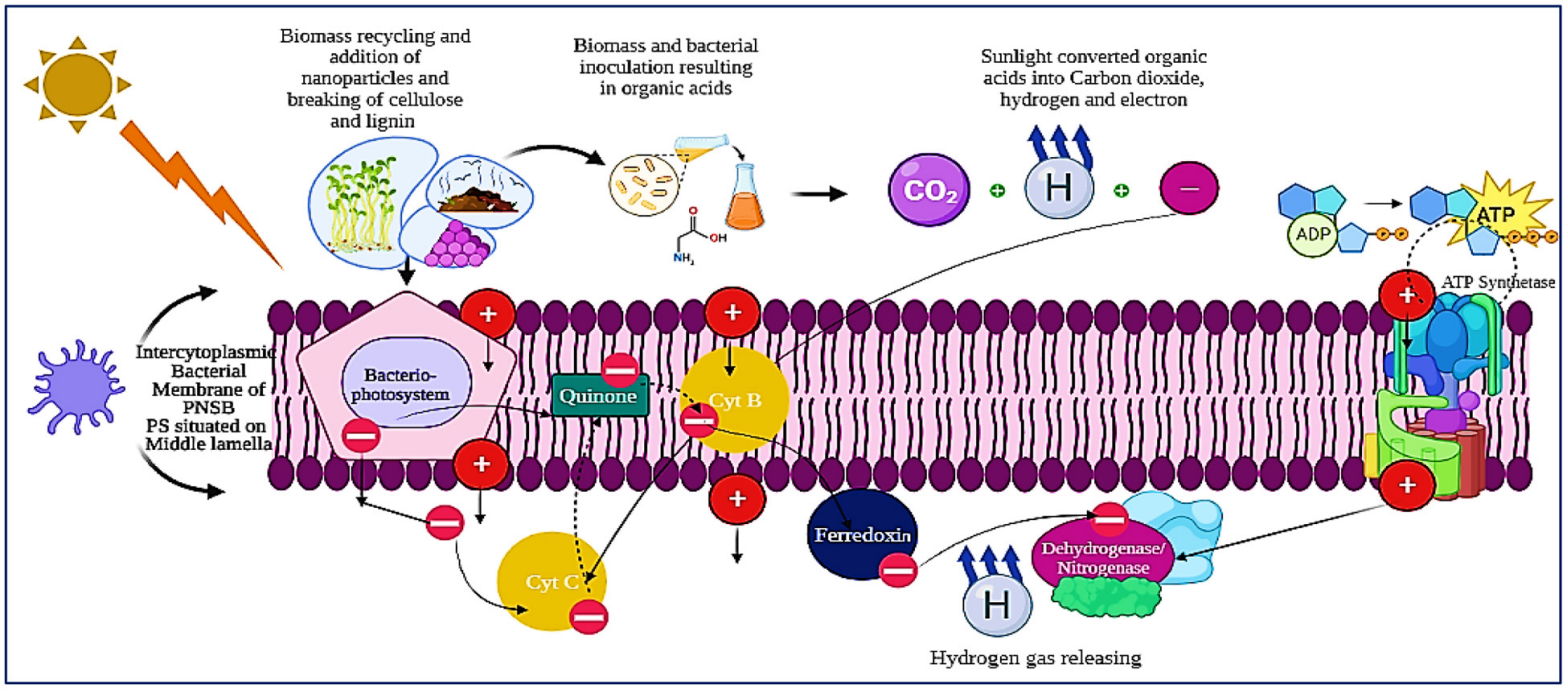
The molecular mechanism of photo fermentation in PNSB and complete degradation of cellulose and hemicellulose into hydrogen.

Nanomaterials are crucial for optimizing biomass conversion to H_2_ and other biofuels by strengthening pretreatment efficiency, enzymatic hydrolysis, and catalytic reforming processes (Bosu et al., 2022). During lignocellulosic biomass conversion, acid-functionalized magnetic nanoparticles (NPs) achieved a cellobiose conversion rate of up to 96%, compared with 32.8% without a catalyst, demonstrating their significant hydrolytic efficacy. Carbonaceous acid-functionalized Fe_3_O_4_ magnetic nanoparticles facilitated the hydrolysis of sugarcane bagasse, achieving a yield of 58.3% at conditions of 160–200 °C and 0.5–2.2 MPa. Multi-walled carbon nanotubes (MWCNTs) boosted the efficiency of enzymatic cellulose hydrolysis to 85–97% and maintained 52–75% activity after six reuse cycles, demonstrating better stability and recyclability. Nickel-cobaltite nanoparticles at a concentration of 1 mM enhanced cellulase activities (endoglucanase by 49%, β-glucosidase by 53%, xylanase by 19.8%), promoting increased release of fermentable sugars. Moreover, nanomaterials enhance enzyme immobilization, protect fermentative microorganisms, and facilitate electron transfer in biohydrogen and syngas production processes, thereby increasing hydrogen yields and enhancing process stability (Bosu et al., 2022; Khoo et al., 2020). In addition to lowering operational expenses without sacrificing catalytic effectiveness, magnetic nanoparticles allow for efficient downstream recovery (Thanigaivel et al., 2022; Bosu et al., 2022). These findings collectively affirm that an optimized nanomaterial dosage (generally in the mM range for metal nanoparticles) and regulated reaction conditions markedly improve biomass depolymerization, sugar yield, and subsequent hydrogen production efficiency, thereby positioning nanotechnology as a crucial facilitator in sustainable biohydrogen biorefineries, as explained in Figure 3.

## 3 Photocatalysis

The term photocatalyst combines the prefix “photo,” denoting light, with the noun “catalyst,” denoting a chemical that accelerates a reaction. In this way, photocatalysts are defined as materials that accelerate chemical reactions in the presence of light (Khoo et al., 2020; He et al., 2020; Li et al., 2025b). Carbon-based, organic, inorganic, and composite NPs are the four main categories. The chemical composition of NPs determines how they behave and perform in different contexts. In addition, NPs feature strikingly different physical and chemical properties from their macro counterparts, which can be exploited in a wide range of contexts, including bioenergy, electronics, medicine, ionic liquids, and consumer goods (Khoo et al., 2020; He et al., 2020; Li et al., 2025b). Furthermore, NPs are crucial for bioenergy production and react more quickly than larger particles. Nanoparticles (NPs) have a high surface-area-to-volume ratio, increasing the number of active sites for various reactions and processes and thereby boosting reaction rates. This is because NPs are extremely finely ground (Basu and Banik, 2024; Vaiano et al., 2018). This process is referred to as photocatalysis All photocatalysts are essentially semiconductors (Wu et al., 2023; Xie et al., 2022). On the basis of the appearance of the physical phase of the reactants, photocatalytic reactions may be divided into two categories.

- Homogeneous photocatalysis: homogeneous photocatalysis occurs when both the semiconductor and the reactant are in the same phase, such as gas, solid, or liquid (Naeem et al., 2021; Chaturvedi et al., 2021).
- Heterogeneous photocatalysis: when the semiconductor as well as the reactant are in different phases, the photocatalytic process is referred to as heterogeneous photocatalysis.

The band gap (E_g_) is the energy difference between the valence band (HOMO) and the conduction band (LUMO). Materials are classified into three primary types: metal (E_g_ < 1.0 eV), Semiconductor (E_g_ < 1.5–3.0 eV), and Insulator (E_g_ > 5.0 eV).

### 3.1 Mechanism for photocatalysis

Prior to discussing photocatalysis, it is necessary to understand the basic behaviors of the excited state of a molecule, which can improve understanding of electron transfer and energy dissipation in semiconductors. The first process is the absorption of a photon by a molecule (in the time scale of 10^−15^ s), where the ground state is lifted energetically to the first excited singlet state (Rahman et al., 2021). Photocatalysis involves reactive oxygen species (ROS) like ^•^OH. Photogenerated *h*^+^ transfer to water molecules oxidizes water to create ·OH radicals and breaks O-H bonds represented as Equations 13–17:


$$\begin{array}{l} {H_{2}O+\;h}_{VB}^{+}\to \;OH+H^{+} \end{array}$$ (13)

**Figure 4:**
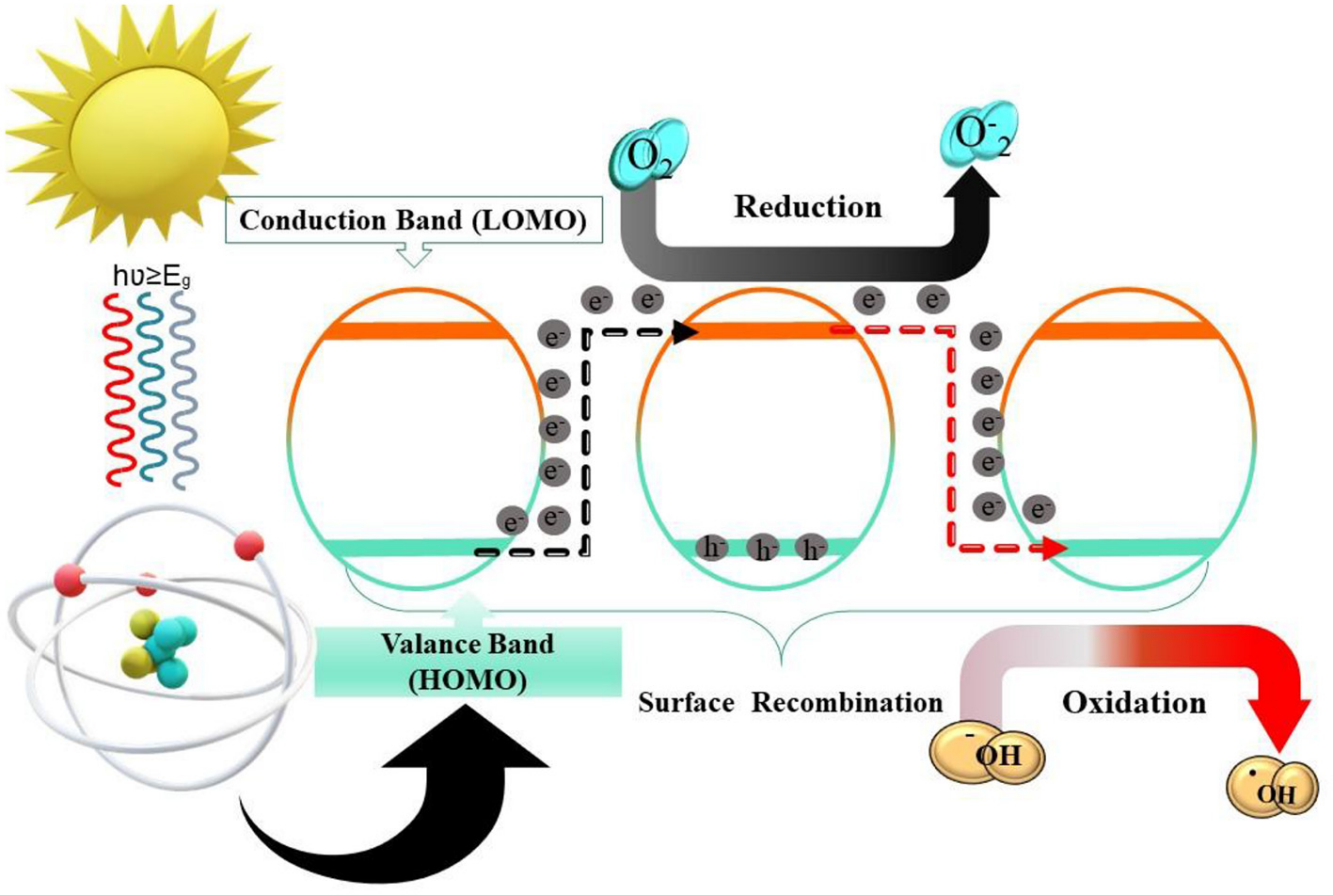
Under light exposure, photons with energy equal to or exceeding the semiconductor band gap (hv ≥ Eg) promote electrons (e^−^) from the valence band to the conduction band, resulting in the formation of electron–hole (h^+^) pairs.

The photocatalytic oxidation of water to O_2_ requires the active species ^•^OH radical. ^•^OH radicals can be adsorbed on photocatalysts or discharged into the bulk solution. The ^•^OH radical may be adsorbed on the surface of photocatalysts to form surface-bound ^•^OH radicals, or it is released into the bulk solution to generate free ^•^OH radicals (Ding et al., 2020). In photocatalytic oxidation processes, the *h*^+^ and surface-bound ^•^OH radicals can only react with adsorbed substrates. Free radicals ^•^OH can react with surface-bound and unbound substrates/intermediates, even weakly adsorbed reactants. Free ^•^OH radicals might oxidize more. Thus, semiconductor-based photocatalysis depends on h+ activating water to create ^•^OH free radicals as illustrated in Figure 4.


$$\begin{array}{l} Photocatalyst+hv\to photocatalyst\left(e_{CB}^{-}\;h_{VB}^{+}\right) \end{array}$$ (14)

$$\begin{array}{l} Photocatalyst\left(e_{CB}^{-}\;h_{VB}^{+}\right)\to e_{CB}^{-}+\;h_{VB}^{+} \end{array}$$ (15)

$$\begin{array}{l} e_{CB}^{-}+2H^{+}\to H_{2} \end{array}$$ (16)

$$\begin{array}{l} h_{VB}^{+}+OH^{-}\to OH \end{array}$$ (17)

## 4 Nano-photocatalysts in bio-hydrogen production via photo-fermentation

**Figure 5:**
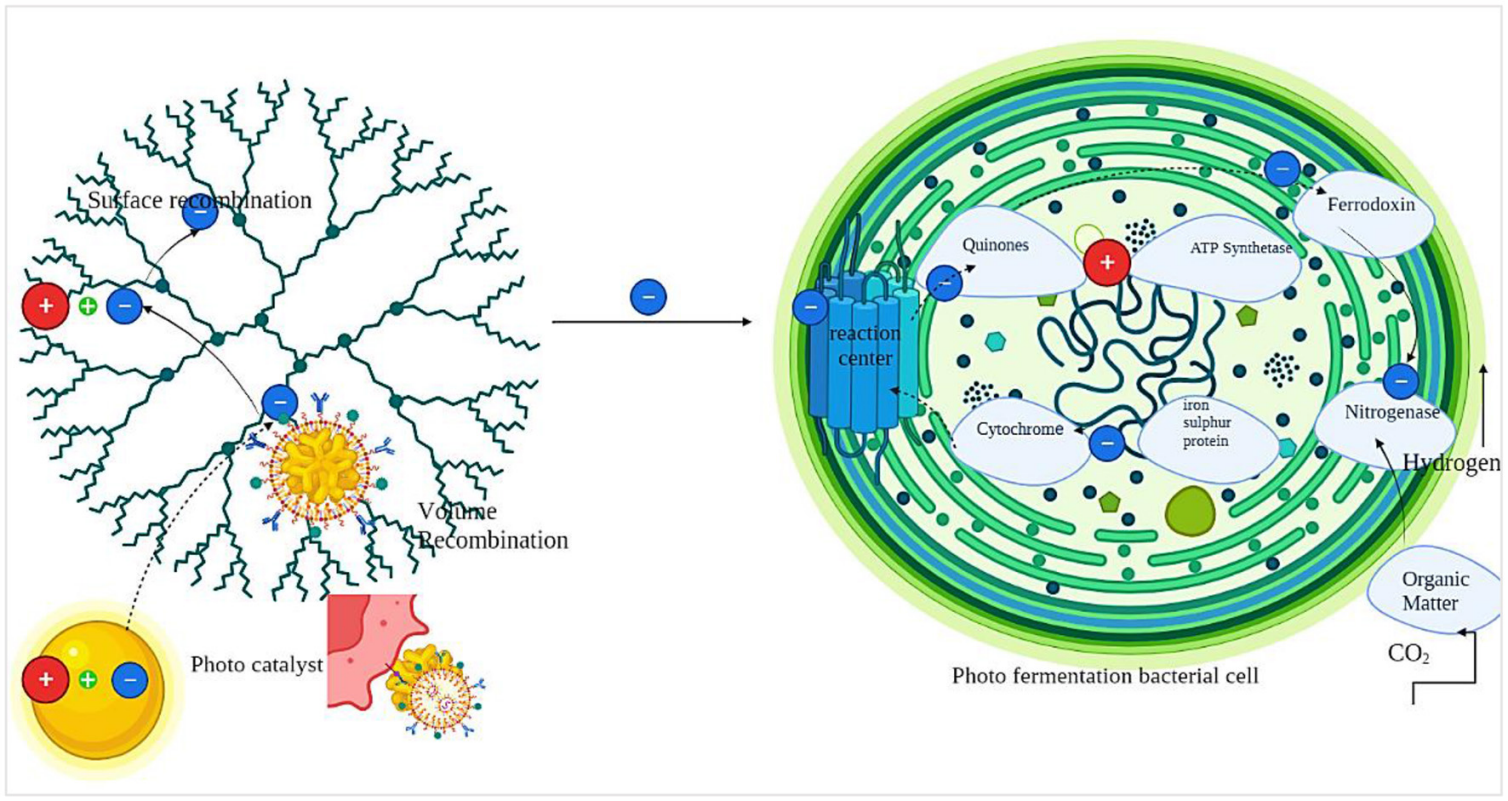
Schematic pathway for bio-hydrogen production via photo-fermentation using nano-photocatalyst.

Nano-photocatalysts have been shown to enhance bioprocess stability and possess unique electrical, magnetic, and surface characteristics. It was also discovered that adding nanoparticles to the culture medium increased biological hydrogen generation. The primary impact of nanoparticles in promoting biohydrogen generation may be due to increased enzyme activity for hydrogen synthesis or enhanced intracellular electron transport (Liu et al., 2023; Nadeem et al., 2020; Tahir et al., 2021). The nano-photocatalyst may also exert significant control over microbial metabolic processes for hydrogen production (i.e., aerobic and anaerobic pathways). The use of a nanophotocatalyst in biohydrogen generation can enhance enzymatic hydrolysis of substrates by immobilizing both substrates and enzymes throughout the conversion process. Furthermore, by directly targeting the hydrogenase enzyme and enhancing electron-transfer efficiency in anaerobic bacteria for improved biohydrogen generation, the integration of a nanophotocatalyst into photofermentation provides a higher specific site density and catalytic activity, as demonstrated in Figure 5 (Acharyulu et al., 2020).

Nanomaterials are crucial for optimizing biomass conversion to H_2_ and other biofuels by strengthening pretreatment efficiency, enzymatic hydrolysis, and catalytic reforming processes (Bosu et al., 2022). In the conversion of lignocellulosic biomass (LCB), acid-functionalized magnetic nanoparticles (MNPs) achieved a cellobiose conversion rate of up to 96%, compared with 32.8% without a catalyst, thereby demonstrating their significant hydrolytic efficacy. Carbonaceous acid-functionalized Fe_3_O_4_ magnetic nanoparticles facilitated the hydrolysis of sugarcane bagasse, achieving a yield of 58.3% at conditions of 160–200 °C and 0.5–2.2 MPa. Multi-walled carbon nanotubes (MWCNTs) boosted the efficiency of enzymatic cellulose hydrolysis to 85%−97% and maintained 52%−75% activity after six reuse cycles, demonstrating better stability and recyclability. Nickel-cobaltite nanoparticles at a concentration of 1 mM enhanced cellulase activities (endoglucanase by 49%, β-glucosidase by 53%, xylanase by 19.8%), promoting increased release of fermentable sugars. Moreover, nanomaterials enhance enzyme immobilization, protect fermentative microorganisms, and facilitate electron transfer during biohydrogen and syngas production, thereby increasing hydrogen yields and improving process stability (Bosu et al., 2022). In addition to lowering operational expenses without sacrificing catalytic effectiveness, magnetic nanoparticles allow for efficient downstream recovery (Thanigaivel et al., 2022; Bosu et al., 2022). These findings collectively affirm that an optimized nanomaterial dosage (generally in the mM range for metal nanoparticles) and regulated reaction conditions markedly improve biomass depolymerization, sugar yield, and subsequent hydrogen production efficiency, thereby positioning nanotechnology as a crucial facilitator in sustainable biohydrogen biorefineries, as explained in Tables 3, 4.

**Table 3:** Application of nanomaterials in PHFP with different lignocellulosic biomass-based H2 production.

Catalyst	Optimal concentration	Process	Biomass	H_2_ Yield	Improvement	References
CoFe_2_O_4_	400 mg/L	PF	Corn stover	379.65 mL	129.0%	(Usman et al., 2024)
CoFe_2_O_4_	400 mg/L	PF	Corn stover	373.30 mL	81.12%	(Usman et al., 2024)
Zn-doped SnO_2_ NPs	150 mg/L	PF	*Paulownia Elongata*	335 mL	~47%	(Tahir et al., 2022)
TbFeO_3_	300 mg/L	PF	Corn stover	268.2 mL	62.62%	(Usman et al., 2025)
ZTFO	200 mg/L	PF	Corn stover	321.28 mL	94.8%	(Usman et al., 2025)
ZTFO/g-CN	150 mg/L	PF	Corn stover	384 mL	132.84%	(Usman et al., 2025)
MWCNTs	20 mg/L	PF	Corn stover	293.84 mL	Two-fold and 16.24%	(Ramzan et al., 2024)
SiC	200 mg/L	PF	*Arundo donax* L	104 mL/g	41%	(Alam et al., 2024)
ZnO	200 mg/L	PF	*Arundo donax* L	98.83 mL/g	37.34%	(Alam et al., 2024)
SnO_2_	100 mg/L	PF	*Arundo donax* L	69.30 mL/g	18.26%	(Alam et al., 2024)

**Table 4:** Mechanistic and emerging materials advances adaptation for improving the PHFP yield.

Mechanistic or materials advances	Feedstock	Inoculum	Parameters	Strategies	Biohydrogen production	References
Integrated dark-photo fermentation	Synthetic lignocellulose hydrolysate	*Rhodopseudomonas pseudopalustris* and *Rhodoplanes piscinae*	pH 7.5	Sequential and co-culture dark-photo fermentations	4.15 mol H_2_/mol sugar	(Zagrodnik and Duber, 2024)
Engineering the microbial-electrochemical interface	Corn stover residues	*Rhodopseudomonas palustris*	(20 mg/L) Co-Fe/BC Incandescent light (3,000 Lux); 30 °C; pH 7	Synergistic of Co-Fe nano biochar composites	HPR and yield by 101.61% and 103.11%, then (CG)	(Tahir et al., 2026)
Photo biostimulation	Succinate, acetate, formate, glucose, sodium glutamate and xylose	*Rhodobacter sphaeroides* KKU-PS1	10.0 and 15.0 klux; 30 °C; 150 rpm	Different light spectra	1,020.3 ± 48.16 mL H_2_/L culture; 1,098.7 ± 47.77 mL H_2_/L culture	(Tiang et al., 2024)
Enhancement of metabolic rate through free electrons Zn-doped SnO_2_ NPs	*Paulownia Elongata*	HAU-M1	Different concentration, Incandescent light (3,000 Lux); 30 °C; 72 h; pH 7	Doping (Zn-doped SnO_2_)	335 mL~ 47%	(Tahir et al., 2022)
Visible light-catalyzed titanium dioxide/activated carbon fiber	Corn stover	HAU-M1	2,000 Lux, pH 6; enzyme loading 200 mg/g TS;	Absorption in the visible spectrum	69.7 ± 2.0 mL/g TS	(Yue et al., 2024)
Increased electron density (Reductive environment)	Corn stover	PNAB	200 mg/L, Incandescent light (3,000 Lux); 30 °C; (72 h); pH 7	Doping, Oxygen vacancy	321.28 mL,94.8%	(Usman et al., 2025)
Delay in e/h pair recombination	Corn stover	PNSB	300 mg/L, Incandescent light (3,000 Lux); 30 °C; (72 h); pH 7	Hetrojection, Delay in recombination electron hole	384 mL, 132.84%	(Usman et al., 2025)
Multidimensional engineering (add pufQ, spbA; add cycY; integrate nif HDK-atpXF)	Acetate and butyrate	*Rhodobacter sphaeroides*	30 °C; 5,000 lux halogen tungsten lamps (12 V, 30 W)	Genetic engineering	61.2 mL/(L·h)	(Zhang et al., 2024)
Synergistic effect of metal Fe and Mo addition	Lignocellulosic biomass	*R. palustris*	15-W LED lamps; 30 ± 1 °C; 150 rpm	Fe and Mo supplementation	48.1 mL/h/L	(de Souza Scotelano et al., 2023)
Addition of Zn^2^^+^ and Mn^2^^+^ ions	Xylose	*R. sphaeroides* HY01	—	Ion's supplementation	219.2 mL/g	(Reaño, 2020)
Selectivity for microbial strains CoFe_2_O_4_	Corn stover	*Rhodopseudomonas palustris* and HAU-M1	Incandescent lamp (190 W/m^2^); 30 ± 2 °C	Selectivity and reusability	421.65 mL (R. palustris); 373.30 mL (HAU-M1)	(Usman et al., 2024)
Constructing *Rhodobacter capsulatus*-ZnO/ZnS hybrid system	Glucose	*Rhodobacter capsulatus* SB1003	Halogen tungsten lamp (13,000 lux); 30 °C	Microorganism-nanomaterial hybrid system	2,479 ± 37 mL/L	(Ghosh, 2022)

## 5 SWOT analysis of photo fermentation via photo nano catalysts

With the shift to renewables and the introduction of waste-to-energy technologies, H_2_ synthesis has become an increasingly lucrative and sustainable option. The large amount of organic waste biomass generated each year has attracted scientists' attention to develop methods for converting these natural biological energy sources into chemical energy (Singh and Das, 2018). This novel approach to biohydrogen production uses photosynthetic bacteria to convert carbon into biohydrogen. Artificial or natural light energy is a crucial component of the entire process. Purple non-sulfur bacteria are the most commonly utilized photosynthetic bacteria (Sharma et al., 2026). Under anoxygenic conditions, photosynthetic purple non-sulfur (PNS) bacteria can use light as an energy source to generate hydrogen from organic acids (such as acetate, butyrate, malate, succinate, etc.) and CO_2_. Consequently, photofermentation offers new avenues for hydrogen production from a range of substrates, including wastewaters and organic acid waste. Depending on the carbon source, light conversion efficiencies as high as 9.3% and hydrogen yields as high as 80% have been reported% (Mishra et al., 2019).

More recently, immobilization of fermentative inoculum has been achieved using various materials, resulting in improved hydrogen production (Mishra et al., 2019). The availability of nanoscale materials (NPs) with suitable physicochemical characteristics has been a huge help. The efficiency of biohydrogen production from biomass, whether microbe- or plant-based, can be significantly affected by nanomaterials and their specific properties (Aboagye et al., 2023). For talks involving the simultaneous conversion of biomass to value-added compounds and hydrogen, photocatalytic biomass conversion to these chemicals was compared to biochemical techniques (Rashid et al., 2024). It is important to answer the following question before bringing photocatalytic technology to market: is it reasonable to expect that photocatalytic technology can be developed to meet the needs of certain applications and problems. Industrial applications raise issues like these to determine whether it is worthwhile to spend resources (money, time, and effort) to address an issue that threatens human health or the environment in order to grow a company. An entrepreneur will request a comprehensive analysis, beginning with a SWOT Analysis, a method for assessing a technology product's strengths, weaknesses, opportunities, and threats in preparation for investment and/or commercialization. It is common practice to view opportunities and dangers as external factors, whereas strengths and weaknesses are seen as internal. With the goal of making it easier to compare with current technologies and bring this technology to an industrial scale, the following points are examined rigorously. To implement the acquired scientific knowledge at scale, a SWOT analysis would be highly useful. In the following sections, we examine the potential implementation of photocatalytic technology by outlining its advantages, disadvantages, market potential, and risks.

### 5.1 Main strengths of photocatalytic technology

Photosynthetic organisms capture incident light using the light-harvesting complex and photoreaction center in the photosynthetic unit. This process turns light energy into chemical energy. In the photosynthetic bacterial system, light is absorbed by pigments such as bacteriochlorophylls (BChl) and carotenoids, which absorb across the wavelength range from 450 to 1,000 nm (Huseynli, 2023). Because of its abundant supply, environmental friendliness, and cleanliness, solar light is considered a renewable energy source with great promise. People are viewing it as a means to address urgent environmental and energy challenges (Fang et al., 2022). The advancement of clean, sustainable energy is crucial for ensuring a stable energy supply and mitigating environmental degradation. Among the many types of clean energy, solar energy is a universal, clean, and sustainable energy source, and its exploitation is a potential means of alleviating energy and environmental problems (Bafaqeer et al., 2023). Due to its electronic excitation and thermal energy characteristics, photothermal catalysis has attracted considerable attention in recent years across a range of industrial and commercial applications. It also promises to make a significant contribution to the acquisition of catalytic surface temperatures. To increase catalytic reaction under photo-radiation and induce charge transfer, photocatalysis (Jiang et al., 2023; Song et al., 2022). Photocatalysis driven by solar light is an efficient technology for eliminating environmental pollutants and for utilizing solar light to produce chemical fuels from CO_2_, such as H_2_, CO, and CH_4_ (Wu et al., 2021; Islam et al., 2023). Specifically, the photocatalytic elimination of harmful and toxic organic species is a photooxidation process driven by solar light, whereas the photoconversion of CO_2_ can effectively reduce CO_2_ to hydrocarbons to alleviate both environmental problems and the energy crisis (Karthikeyan and Velvizhi, 2024).

Biological routes of hydrogen production are considered carbon-neutral methods and offer potential advantages over thermochemical and electrochemical processes. Operating with ambient pressure and temperature, while requiring minimal energy input, can be achieved by utilizing a diverse range of biologically flexible renewable resources (Feijoo et al., 2019). Nanomaterials are increasingly used in various applications as substitutes for bulk chemicals (Ibrahim et al., 2023). The photocatalyst is suitable and capable in the reaction process with methanol and FFA under light irradiation. Not only that, it is environmentally friendly, reusable, and yields high levels, which ensure the success of new alternative energy production (Mashuri et al., 2020). Photocatalyst has been widely used in the applications of wastewater treatment (Zhang et al., 2022), bacteria disinfection (Pellegrino et al., 2019), hydrogen production (Wang et al., 2022), and CO_2_ removal (Shaikh et al., 2023). PNSB possess the ability to perform a diverse range of beneficial actions, including: N_2_ fixation, phosphate solubilization, heavy metal remediation, CH_4_ emission mitigation, and CO_2_ sequestration (Huu et al., 2022; Karimi-Maleh et al., 2022b). Nanotechnology is a promising solution to critical environmental challenges due to its exceptional performance and effectiveness across diverse applications across multiple disciplines (Karimi-Maleh et al., 2022a; Huang et al., 2017).

PNSB can transition among multiple metabolic modes, including photolithoautotrophy, photo-organoheterotrophy, chemo-organoheterotrophy, and chemo-lithoautotrophy. The specific mode it adopts depends on the types of electron donors and acceptors that are available in the growth medium (Iervolino et al., 2019). The use of photocatalysts such as TiO_2_ and ZnO is limited by the requirement for ultraviolet (UV) activation, as their bandgap energies are approximately 3.2 eV. Consequently, less than 5% of the solar spectrum possesses enough energy to activate the photocatalyst. Additionally, the rapid recombination rate of the electron-hole pairs generated poses another limitation (Arif et al., 2023). Therefore, enhancing photocatalytic performance and activation under visible-light irradiation often involves modifying photocatalysts by introducing metal or non-metal elements or by combining them with another semiconductor. The particle size and morphology of nanoscale photocatalysts pose a challenge for the practical implementation of water and wastewater treatment, as particles must be effectively removed or recovered post-treatment. To address this limitation, researchers have suggested using photocatalytic membranes or other materials that support photocatalysts (Shen et al., 2022). Tactics for modifying photo-nano catalysts include introducing defects/vacancies, alloying, adjusting interfacial structures, and fine-tuning catalyst morphology (Kis et al., 2022). Various synthetic techniques, such as doping, facet engineering, and heterojunction, have been proposed to control the valence band maximum and conduction band minimum of particle materials (Iragavarapu et al., 2023).

Photofermentation, driven by phototrophic PNSB, converts various organic substrates, including short-chain organic acids, into electron donors for molecular hydrogen production and operates more efficiently under anaerobic conditions. One of the first photosynthetic organisms on Earth, PNSB are red or purple oxygenic and anoxygenic bacteria. PNSB uses electron and proton transfer processes to convert light energy into metabolic energy for survival. Photosynthesis begins when light-harvesting pigments absorb light and transfer electronic excitation energy to the reaction center (Iervolino et al., 2019; Matyakubov et al., 2022; Budiman and Wu, 2018). Photocatalytic conversion of biomass into high-value products is a potential alternative to traditional catalytic methods (Haarstad et al., 2006). Organic waste, such as waste-activated sludge, algal biomass, cellulose-based biomass, starch-based biomass, food waste, and wastewater, is commonly used as a feedstock for fermentative hydrogen production. Natural or human-produced, these compounds are rich in key components (carbon, nitrogen, and phosphorus), inexpensive, and abundant, making them suitable substrates for biological hydrogen synthesis (Al Azad and Mohamad Lal, 2023). Biodegradation of waste involves methane (CH_4_), CO_2_, H_2_, and O_2_. However, N-chemicals, volatile organic compounds (VOCs), and others, including CO and H_2_S, are also formed (Bianchi et al., 2020). Agro-based waste is efficiently converted into nutritionally valuable products by PNSB (Djellabi et al., 2021). Photocatalysis's non-chemical use is a key advantage over other advanced oxidation technologies. In contrast to conventional advanced oxidation processes (AOPs), photocatalysis does not require H_2_O_2_, a substantial cost. This characteristic enables continuous environmental remediation techniques, such as self-cleaning materials, to reduce surrounding chemical and microbiological pollution (Samer, 2023; Vasiliadou et al., 2018).

### 5.2 Shortcomings of photocatalytic technology

In anaerobic conditions with light, PNSB can grow heterotrophically and create biohydrogen from volatile fatty acids, including acetic, propionic, butyric, and valeric acids. However, biohydrogen generation requires specific light intensities (3–10 klux), wavelengths (400–1,000 nm), temperatures (31–36 °C), and pH (6.8–7.5) (Wang et al., 2019). Based on the application, pH can greatly impact bioelectrochemical systems (BES) performance. For optimal biohydrogen synthesis, pH must be between 6.5 and 7 (Ghosh et al., 2017; Gupta et al., 2024). Temperature affects the growth and metabolism of Anoxygenic phototrophic bacteria and the generation of current in bioelectrochemical systems (BES) (Li et al., 2016). PNSB can produce H_2_ only in anaerobic, heterotrophic environments under solar or artificial light (Mahlangu et al., 2023). Photocatalytic reactions use photon energy rather than thermal energy; they can be conducted at ambient pressure and temperature (Deng et al., 2023). Semiconductor photocatalysts exhibit poor photocatalytic performance due to photogenerated charge-carrier recombination (Weon et al., 2019; Chen et al., 2020).

Industry needs constant processing because photocatalyst deactivation hinders continuous use (Aruoja et al., 2015). Regrettably, photocatalytic materials undergo surface deactivation after a specific period of use, necessitating cessation of activity and implementation of a regeneration procedure. Deactivation is commonly associated with excessive adsorption of contaminants and/or the formation of organic and inorganic by-products. The effect can be more pronounced if the accumulated pollutants or byproducts exhibit greater resistance to reactive oxygen species (ROS) generated by the photocatalyst on its surface (Toepel et al., 2023). Most research on aldehyde degradation focuses on acetaldehyde and formaldehyde, which are the most extensively studied compounds in the breakdown of volatile organic compounds (VOCs). Due to the difficulty of degrading aromatic VOCs such as benzene, toluene, ethylbenzene, and xylene, and their tendency to deactivate catalysts, a significant amount of research (up to 28.7%) has focused on using visible-light photocatalysis to degrade these pollutants (Aruoja et al., 2015). In general, the deactivation process is influenced by the effectiveness of photocatalysts in oxidizing specific pollutants at a particular concentration.

The proliferation of metal-based nanoparticles in various applications has raised concerns over their environmental impact. The absence of high-quality nanotoxicity data for test species relevant to the environment, along with the lack of physicochemical characterization of nanoparticles (NPs), significantly impedes risk assessment (Khan, 2023). Nitrogenase, the primary biocatalyst in PNSBs, catalyzes hydrogen production. Oxygen has a highly detrimental effect on nitrogenase, rendering it permanently inactive (Wu et al., 2020). Therefore, by preventing oxygen and nitrogen from entering the reaction environment, the production of biohydrogen in PNSBs via photofermentation is improved. A major constraint in implementing photocatalytic processes for full-scale water treatment is the preparation of photocatalysts, which significantly impacts process costs, particularly compared with homogeneous photo-driven advanced oxidation processes (AOPs) (Zhang et al., 2021). The challenges of intricate structural design, complex synthesis, inconclusive testing, and prohibitive costs must be resolved before photochromic materials can gain commercial acceptance (Bianchi et al., 2019; Sales et al., 2021).

### 5.3 Marketing opportunities and threats

The primary potential is in harnessing the advantageous and distinctive properties of photocatalysis to address environmental and human health issues. This includes developing marketable goods and solutions. Individuals who possess exclusive expertise and technology in photocatalysis, specifically those capable of addressing the limitations of the process, such as developing materials with exceptional activity in the visible range, creating stable and durable supported catalytic systems, implementing cost-effective and efficient catalyst regeneration processes, and adhering to sustainability standards outlined by LCA/Circular Economy, have the potential to seize various opportunities. Photocatalysis can serve as a temporary replacement for existing systems in some situations. Specifically, during bright periods or in locations with prolonged sunlight, solar photocatalysis can effectively replace chemical AOPs throughout the treatment system. Photocatalysis possesses numerous distinctive characteristics that have the potential to be enhanced and used to specific real-world applications. For instance, the process of converting certain harmful substances into valuable products using photocatalysis. The integration of photocatalysis helps mitigate the prevalent problem of fouling in ultrafiltration membrane systems (Buffi et al., 2022). The application of photoactive nanoparticles to coat building tiles is a highly desirable approach for creating self-cleaning materials and has attracted significant attention from industry (Skillen et al., 2022).

Photocatalysis needs to become at least one order of magnitude more efficient, a persistent hurdle that remains unresolved despite extensive research efforts (Jiang et al., 2023). The use of customized catalysts, rather than commercially available ones, can have a significant impact. The aim of these improvements is to enhance the efficiency of radical production, specifically by increasing the quantum yield of hydroxyl radical formation. Additionally, these modifications should enable the use of visible-light photonic energy, extending beyond the UV region, thereby enabling solar treatment. Industrial-scale photocatalytic technology may encounter several challenges, which is to be expected. The proliferation of alternative developing technologies could potentially restrict the marketing of photocatalytic devices. The efficacy of a photocatalytic product may diminish over prolonged usage. Photocatalytic activity can be compromised when initial conditions are altered, such as when wastewater composition changes, which may impede photocatalytic activity due to specific contaminants. Market success is contingent on establishing trust, which requires demonstrating one or more highly effective applications of photocatalysis.

## 6 Technology readiness level (TRL)

Assessing the potential of photocatalytic biomass reforming is difficult due to the relatively recent development of the technology. To accomplish this, it is necessary to ascertain the current and desired field conditions and subsequently calculate the difference between them. Additionally, it is necessary to identify the factors that may affect technological advancement and the specific motivations that may propel it, such as commercial interests. In this discussion, we will provide a concise overview of photocatalysis as a method and technology for converting biomass into electricity. This involves identifying and classifying obstacles, evaluating the TRL of biomass photocatalytic conversion, and developing a roadmap outlining a strategy to promote future technical advancement. Most papers on biomass conversion focus on research findings or summarize previously published work. However, there is limited emphasis on the technological potential and practicality of implementing the process in real-world applications (Nishiyama et al., 2021; McCullagh et al., 2011).

**Figure 6:**
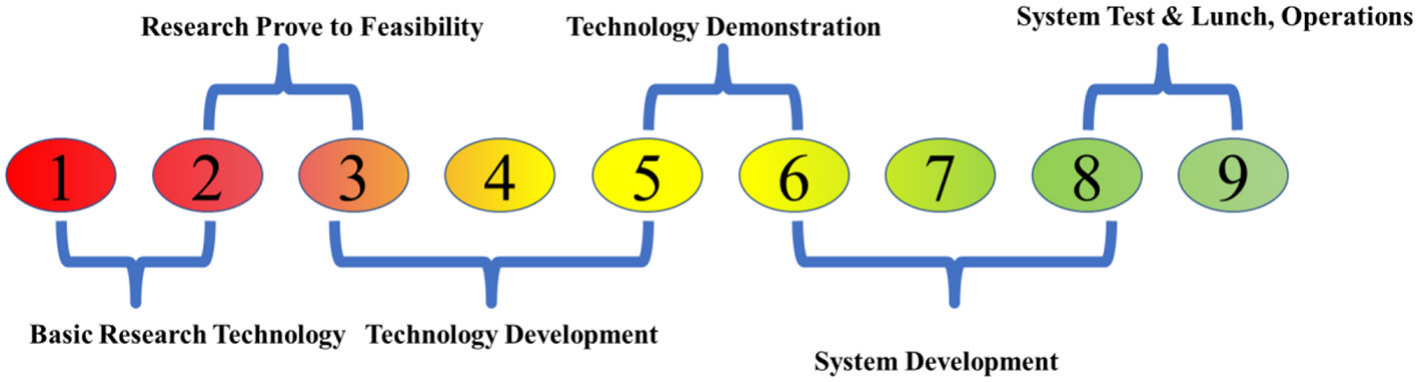
Technology development to implementation pathway for photocatalytic systems, depicting the transition from laboratory-scale research to pilot validation and ultimately full-scale industrial deployment, with emphasis on scalability, efficiency, and practical applicability.

The TRL can be represented on a numerical scale from 1 to 9, with a concise explanation for each level, as illustrated in Figure 6. The TRL scale is a valuable instrument for evaluating novel technologies, such as photocatalysis and photo fermentation. The various stages of application encompass the TRL scale. A TRL of nine indicates that the technology has reached a high level of maturity and is ready for widespread implementation. Only a small number of photocatalytic reactions, with self-cleaning surfaces being the most probable, support this claim (Nishiyama et al., 2021). The TRL typically ranges from 1 to 6, with environmental remediation being more advanced than chemical synthesis or energy production (Ashraf et al., 2023). The TRL of photocatalytic processes often reflects the chemical complexity and intricacies linked to these applications. The non-selective nature of ROS enables more efficient removal of impurities from air and water than the production of an energy carrier (e.g., H_2_) or a specific bioproduct (e.g., value-added chemicals). Furthermore, the process may be hindered by competing reactions, such as electron removal or the oxidation of reaction products, thereby reducing its feasibility. The TRL for the photocatalytic reformation of biomass ranges from 1 to 3. Additional subcategories may be included in the TRL scale for principal applications, such as H_2_ generation, bioproduct production, and combination systems that involve simultaneous energy and value-added connections. When evaluating the TRL status of a technology, it is crucial to ensure that the specific level requirement (e.g., TRL 5) is consistently met and, on a case-by-case basis, shown to be justified. Photocatalytic technologies can be evaluated based on their current and desired states (Ashraf et al., 2023). The production of H_2_ has reached TRL 3, indicating the highest level of advancement in the biomass process. Nevertheless, this value remains subject to alteration based on the particular circumstances. Raw biomass, such as lignocellulose and lignin, is currently in the early stages of development (TRL 1–2). In contrast, laboratory experiments have already yielded successful results using other feedstocks, such as sugars and organic acids. These substances have been used for hydrogen generation even before the introduction of photocatalysis. Biomass is accessible as a more cost-effective substrate; the photo reforming process for H_2_ production can also draw on existing knowledge of photocatalytic water splitting and H_2_ generation from alternative sources (Rumayor et al., 2022).

Using the TRL scale or comparable methodologies, it is possible to evaluate the technology and assess its potential for new applications. An illustrative instance is the life cycle analysis (LCA) conducted on the photocatalytic reformation of waste materials, such as glycerol, to generate H_2_. This analysis demonstrates the environmental consequences and the process's probable viability for evaluation purposes. Two reactor configurations were evaluated for irradiation: synthetic irradiation with LEDs and direct solar irradiation with a parabolic trough collector. Several operational scenarios (Sc) demonstrated the challenges associated with doing this particular research, as well as the promising capabilities of photocatalysis. The direct solar irradiation, such as Sc. 2-PV-Solar and Sc. 2-Wind, had the lowest global warming potential (GWP) of less than 0.4 kg CO_2_-eq. In an alternative scenario, renewable energy was used exclusively for mixing and stirring. Thus, photocatalytic technology was designed to produce environmentally friendly H_2_ at a relatively slow rate (e.g., approximately 5 × 10–4 kg H_2_ per hour) with a GWP similar to that of other sustainable methods, such as water electrolysis. This assertion is significant in photocatalysis research. Significantly, this work is among the pioneering studies that utilize SWOT methodology to investigate photocatalytic H_2_ generation. Moreover, the scenarios oversimplify an intricate reality. Hence, it is imperative to continue conducting these investigations to identify and address obstacles arising from prospective research domains.

The issues and difficulties related to biomass PR, namely the alignment between the oxidation reaction and its products, as well as hydrogen generation, are connected to the (i) catalyst, (ii) reactor process, and (iii) light in a broad sense. However, there are other distinct obstacles unrelated to water-splitting and/or CO_2_ reduction. To evaluate the technology, it is crucial to define a feasible objective for an effective procedure. The assessment can be based on the specific biomass feedstock, the desired products, the attainable product yields, and the overall process efficiency required to achieve those yields. Yield and irradiation source are crucial for the photocatalyst, as the production of H_2_ and value-added chemicals from biomass remains low and far below the levels required for pilot-scale facilities. The enhancement in crop production and effectiveness relies on the inherent properties of the photocatalyst, namely its electronic structure's capacity to be stimulated by sunlight. For almost two decades, there have been documented instances of photocatalysts that use visible light to perform both water splitting and energy conversion, with varying degrees of efficiency. For a photocatalyst to be considered viable for commercial use, it must not only be capable of producing large amounts of goods but also meet the necessary requirements for practical application. With the exception of a modified form of titanium dioxide (TiO_2_) called Kronos, no other photocatalysts are currently available. To carry out a process at large scale, it is necessary to have access to a photocatalyst in quantities of kilograms or even tons. The commercially available TiO_2_ product from Evonik operates in conjunction with UV radiation at approximately 385 nm (McCullagh et al., 2011; Ashraf et al., 2023).

**Figure 7:**
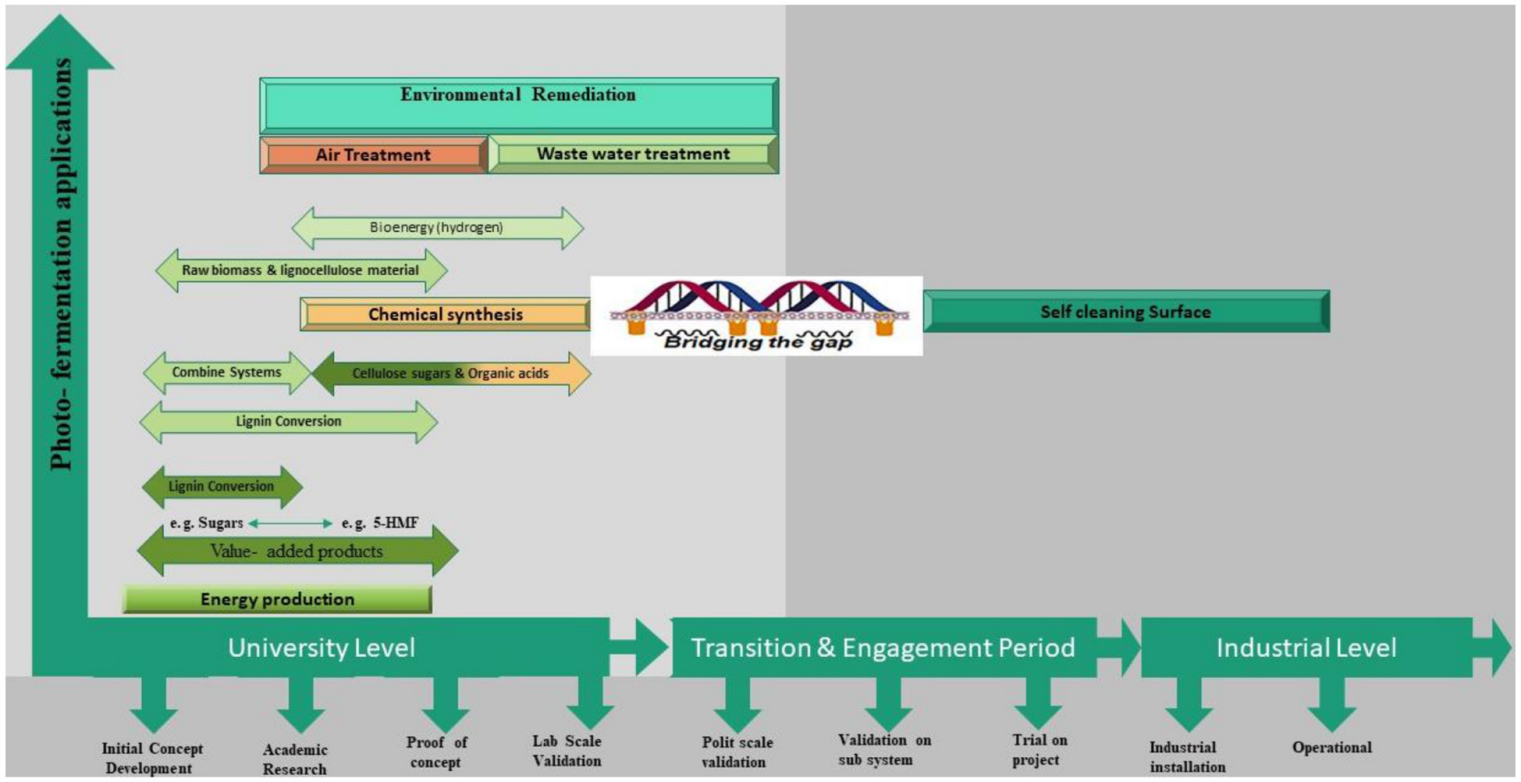
TRL scale for photocatalytic fundamentals to practical applications.

Aside from the invention of catalysts, their use is of utmost importance. The reactor is responsible for optimizing the active catalyst surface area, the intensity and dispersion of light, mass transport, and product separation. Nevertheless, these concerns constitute crucial challenges in biomass conversion that must be addressed during prototype development or reactor design (Rumayor et al., 2022; Sundar and Kanmani, 2020). The TRL scale (Figure 7) illustrates the challenges of overcoming technological obstacles, including validating at the pilot scale, conducting large-scale trials, and engaging industry participants. Moreover, it is feasible to proceed even if the implementation of photocatalytic biomass conversion has a lower TRL compared to water treatment. Specifically, the TRL for biomass reformation and environmental remediation is 3 and 6, respectively. It is necessary to establish priorities that account for both impediments to technology transfer and basic research. Furthermore, supplementary rewards for technological advancement are sometimes overlooked or not typically linked to other photocatalytic applications. The adoption of zero-emission objectives has fundamentally transformed the approach to renewable energy plans and will have a lasting impact on the future energy landscape. These considerations are also applicable to numerous photocatalytic applications, particularly those involving the conversion of biomass and/or waste and the reduction of CO_2_, both of which are currently of utmost importance.

The following aspects are crucial in formulating a strategy for future implementation:

Catalyst development that facilitates technical advancement outside the confines of the academic laboratory. The primary objective of every photocatalytic application is to evaluate activity under visible and solar irradiation, while accounting for other essential characteristics. Prior to proceeding, it is imperative to assess the economic and commercial viability of large-scale synthesis and implementation of the technology. Additionally, it is crucial to ensure that the operation can be performed either in thin-film form or with a robust support structure. To convert biomass, the catalyst must produce hydrogen in the millimole range with a photonic efficiency exceeding 50%. Furthermore, in order for solar hydrogen generation to be economically feasible, it is necessary to obtain a solar-to-hydrogen efficiency of 5%−10%.

Reactor technology must be given the same level of importance as research areas. This involves using existing modular reactor systems (e.g., electrolyzers and fuel cells) to develop modular photocatalytic units that demonstrate functionality across a range of scales, particularly for advancing pilot-scale units. Furthermore, the advancement of irradiation systems must exceed conventional laboratory standards, such as those using Xe arc lamps, by adopting a dual strategy that integrates solar and sustainable artificial irradiation. However, solar irradiation does not include physically exposing the catalysts to sunlight. Instead, it entails designing systems that simulate and enhance the distribution, exposure, and concentration of solar photons on the catalyst surface. Contemporary LED systems can use renewable energy sources, such as wind power. Furthermore, it is imperative that such a system seamlessly integrates with the existing infrastructure to effectively use H_2_ as an energy carrier.

**Figure 8:**
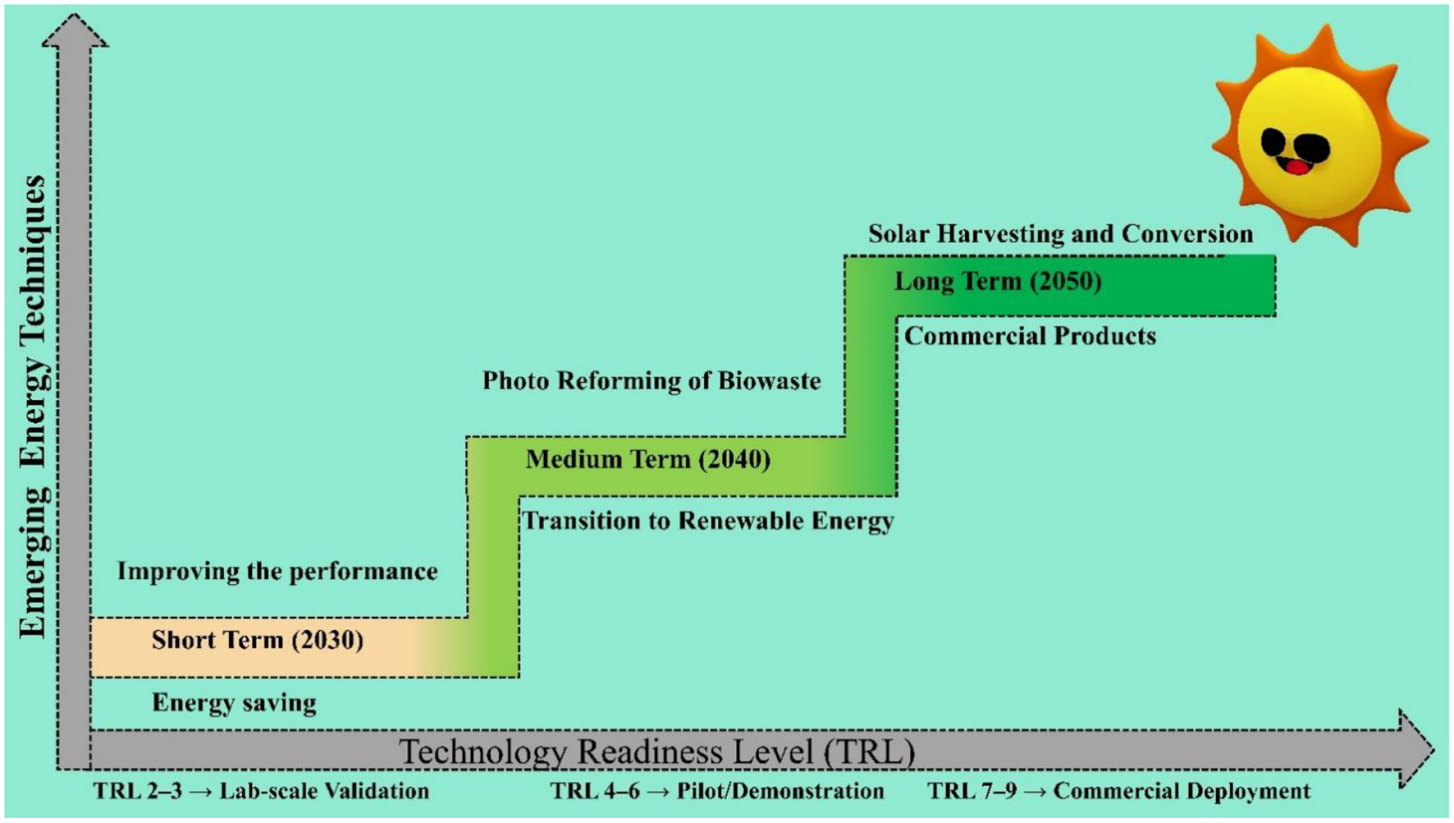
Roadmap toward commercialization of biohydrogen from biological pathway.

Precise and inclusive measures are necessary as evaluation instruments. An uncomplicated metric would be preferable, but it overly simplifies a problem with multiple variables. An evaluation must consider multiple criteria in order to be thorough and accurate. An H_2_ value, when combined with photonic efficiency, can be used to calculate the photocatalytic space-time yield. This yield accounts for conversion rates and reactor-specific metrics, such as scale and electrical power. Furthermore, the metrics should encompass the assessment of the photocatalytic reforming of biomass with respect to energy balance and life cycle. In addition, the evaluation considers a scientific assessment of the cost (expressed in €/kg H_2_) and the environmental impact (measured in CO_2_-eq/kg H_2_), relative to industry standards. Prioritizing development effort based on specific characteristics. Photocatalysis research continues to prioritize catalyst development. There is a need to enhance the TRL of industry-related initiatives illustrated in Figure 8. This encompasses reactor technology, the formulation of appropriate metrics, catalyst production, and standardized testing. To assess research goals and facilitate the integration of basic research with industry, it is crucial to conduct techno-economic analyses and life-cycle assessments of the entire systems involved in the photocatalytic reforming of biomass. It is crucial to determine the life-cycle inventory for the photocatalytic reforming of biomass to optimize the results of the system analysis.

## 7 Life cycle assessments of biohydrogen production

The life cycle assessment (LCA) method is a well-established approach for examining a product's environmental impacts from cradle to grave, encompassing all phases of manufacture, use, and recycling (Sivagurunathan et al., 2017). Using LCA methods, multiple studies have assessed the ecological consequences of biohydrogen production with respect to fossil fuel consumption, GHG emissions, and other environmental metrics (Djomo and Blumberga, 2011). Biohydrogen is currently produced commercially only from lignocellulosic biomass (Nishiyama et al., 2021); hence, most LCA studies have relied on data from pilot- or lab-scale experiments or process simulation models (Ochs et al., 2010; Sambusiti et al., 2015). The energy consumption and environmental impact of producing biohydrogen from three different feedstocks, wheat straw (WS), sweet sorghum (SS) stalk, and sweet potato peels (SPP), were studied. In contrast to SPP, which can be hydrolyzed directly, agricultural wastes (WS and SS) require an energy-intensive pretreatment step prior to sugar hydrolysis. Because the feedstocks had varying chemical compositions, the quantities of enzymes and reagents used differed, even though the other unit processes were identical. Adding the co-product credit had a significant effect on the outcomes. Among the feedstocks tested, SPP had the least negative environmental impact, followed by WS and SS. Resource depletion and greenhouse gas emissions followed similar patterns, with SPP identified as the optimal hydrogen feedstock. Hydrogen produced from these three feedstocks had emissions that were 52.5%−56% lower than those of diesel and 54.5%−57% lower than those of methane reforming, respectively. Since SPP did not necessitate pre-treatment, it exhibited the highest energy ratio (2.67 compared to 1.54 and 1.4 for WS and SS, respectively) among the three feedstocks. Because bioreactors consume substantial power during fermentation, hydrogen production accounts for the majority of greenhouse gas emissions (Rajesh Banu et al., 2020).

Even more notably, sugarcane had significantly lower emission reduction values (23.74–31.06 g CO_2_ eq./MJ H_2_; 74 and 87% lower). When comparing the energy consumption and greenhouse gas emissions of the distribution system with those of the agricultural process for potato peels, the distribution system performed better, assuming sugarcane transportation from Brazil. It helped reduce emissions by 7% because peels are regarded as waste. Nevertheless, an LCA study found that hydrogen produced by fermenting potato peels was far less effective and had a greater environmental impact than hydrogen produced by steam reforming (Touloupakis and Torzillo, 2019). These outcomes were attributed to the inputs, primarily phosphorus, used in the fermentation process. At the same time, this study's hydrogen output was about a third of what Ahmed et al. (2021) reported, thereby highlighting the importance of the assumptions employed in the process's computations and simulations. Dark fermentation of microalgae produced hydrogen; however, the resulting greenhouse gas emissions were excessive and unsustainable, weighing in at 5–6 kg CO_2_ eq./MJ. The majority of these emissions, over half, were caused by the growth and raising of microalgae (Genç and Koku, 2024). Meanwhile, emissions were found to be highly sensitive to the energy mix used for electricity generation. Also, in scale-up scenarios involving optimized cultivation using advanced techniques, emissions were reduced by 14%. Further, on accounting the CO_2_ absorbed, the total emissions were found to be negative (−584 to −406 g CO_2_eq./MJ H_2_). These results further emphasize the importance of selecting appropriate assumptions, the scale of operation, and the data used in performing an LCA. At the same time, emissions were highly sensitive to the electricity generation mix. Furthermore, a 14% decrease in emissions was observed in scale-up scenarios that accounted for improved cultivation practices employing sophisticated techniques. The total emissions were determined to be negative (−584 to −406 g CO_2_eq./MJ H_2_) after taking into consideration the CO_2_ that was absorbed. These findings further highlight the importance of selecting appropriate assumptions, operational scales, and data for an LCA.

## 8 Techno-economic analysis of catalytic biohydrogen production

One well-known method involves the efficient and economical synthesis of hydrogen gas from biomass or organic wastes using biological processes mediated by the enzyme hydrogenase. Dark fermentation and photo-biological H_2_ generation are two main categories of biomass conversion (Touloupakis and Torzillo, 2019; Cristóbal et al., 2018). Evaluating the process's economic feasibility and identifying opportunities for improvement are the focus of a techno-economic analysis (TEA) of catalytic biohydrogen generation from biomass. A TEA of this process accounts for feedstock cost, process efficiency, and catalyst cost, among other important considerations (Ren et al., 2016). Photo fermentative hydrogen production offers a sustainable energy solution by utilizing inexpensive raw materials and sunlight. Although numerous successful laboratory and pilot-scale experiments have demonstrated light fermentative production, the overall economic feasibility of this process remains unproven. The cost of fermentative hydrogen production from a specific picture was evaluated using an existing outdoor pilot-scale (20 L) photobioreactor. The annual capital cost was determined to be USD 124, covering both equipment and land. The operating cost was estimated at 42 USD per year, covering maintenance, energy, and feedstock costs. The precise cost of hydrogen generation was determined to be 2.7 USD/mol (1,362 USD/kg H_2_), and 395 USD/kg H_2_ when the cost of equipment is subsidized (Hsu and Lin, 2016b). Cristóbal et al. (2018) identified several crucial sources of uncertainty that should be accounted for in sensitivity analysis. These include the cost calculation method, revenue, utility costs, process variables, feedstock quantity, and transport. One source of uncertainty in sensitivity analysis is the cost estimation method, which can lead to a ±30% divergence in the inflation or deflation of the suggested capital cost (Bundhoo and Mohee, 2016).

### 8.1 Technical barriers

Higher production costs and lower production efficiency relative to alternative approaches are among the challenges impeding the commercialization of H_2_ production (Kumar et al., 2017). Despite the steady advancement of technology, the primary stage of fermentative H_2_ production remains expanding (Ghimire et al., 2015). To increase H_2_ yield and generation rate, various genetic and fermentative strategies must be implemented. The strains required to produce H_2_ are engineered. The economics of the H_2_ production process can be improved through process integration. Higher pretreatment costs for inoculums and increased energy consumption in the DF process affect commercialization. It is imperative to assess the economic and technological viability of DF before commercialization to prevent large-scale process blockages (Argun et al., 2016; Hosseinzadeh et al., 2022). The H_2_ yield in DF is limited to 4 mol H_2_/mol glucose, which is still a significant technical challenge (Soares et al., 2020). The production of biological H_2_ is a well-known barrier. However, researchers may overcome this obstacle by generating competitive H_2_ production (Show et al., 2019). Optimization of the entire process is necessary for any H_2_ process to be commercialized. With its 15 kg CO_2_-eq/Kg hydrogen emissions, the fermentation process produced the fewest greenhouse gas emissions in terms of potential environmental effects (Aslam et al., 2018).

### 8.2 Economic barriers

The DF process in biological H_2_ synthesis is highly costly, and most research is conducted at a laboratory scale. The financial and technological hurdles that impede the commercialization of several biochemical conversion processes for hydrogen production. Thus, to become a more competitive and viable technology, the PF still requires large-scale investigations to overcome techno-economic constraints (Yukesh Kannah et al., 2021). Major economic hurdles include developing low-cost photo-bioreactors and optimizing photosynthetic reactions in the biophotolysis process (Chandrasekhar et al., 2015). In their study on H_2_ production using anaerobic membrane bioreactors, Aslam et al. (2018) found that the primary financial obstacles are higher operating and installation costs, which in turn result in lower hydrogen yields (Nikolaidis and Poullikkas, 2017). Since biological H_2_ generation is an expensive process, the economic analysis of photobiological H_2_ production is heavily predicated (Hsu and Lin, 2016a). Because of its low H_2_ production rate, the photobiological method is unsuitable for industrial-scale applications. H_2_ generation is enhanced through the integration of multiple secondary processes to the primary process. To efficiently produce hydrogen, the pF is combined with secondary processes, such as photobiological processes, microbial electrochemical cells, and microbial fuel cells. Secondary processes are economically valuable because they generate more energy (Vinardell and Valderrama, 2026). An estimated 2.5–2.8 USD/kg was spent on integrated dark and photo fermentative H_2_ production (Nikolaidis and Poullikkas, 2017). According to Sharma and Kaushik (2017), the generation of H_2_ in DF and PF cost approximately 3.70 and 18.70 USD, respectively. Due to its higher cost, dark-fermentative H_2_ production has seen limited commercialization relative to natural gas reforming (Hsu and Lin, 2016a).

## 9 Challenges and future recommendations

The biomass methods discussed in the previous section offer potential for H_2_ production, but they face constraints that may limit their application. Various nanoparticles have been added to the fermentation medium to enhance H_2_ production. Given limited research on nanomaterial requirements, future research should focus on nanoparticle dosage to maximize bioavailability while minimizing toxicity to fermentation microbes. H_2_ generation requires metalloenzymes (hydrogenases) with appropriate levels of metallic nanoparticles to maintain catalytic sites and minimize feedback inhibition. Nanoparticles made from low-cost biomaterials with strong physicochemical properties may prevent lysis and degeneration of fermenting microbial cells. Nanoparticles within the microbial fermentation population significantly increased H_2_ production. Analyzing the effects of nanoparticles on bacterial community structure and dynamics helps identify beneficial H_2_ producers. H_2_ has been produced only in batch mode using nanoparticles. Continuous operation studies are needed to identify technical hurdles to large-scale commercial applications. To facilitate microbial and enzymatic binding in bioreactors, sheets, nanotubes, or composite materials can enhance H_2_ generation. Nanoparticle toxicity in fermented residues warrants investigation. Future research on recycling leftover nanoparticles may solve this problem.

Some of these are highlighted below:

1. Currently, H_2_ synthesis via biological processes is more expensive than alternative biofuels. Furthermore, a full techno-economic analysis is necessary to map the effects of various parameters on the final H_2_ price (Vinardell and Valderrama, 2026).
2. Additionally, the Analytical Hierarchy Process (AHP) allows for optimal decision-making in immobilized systems. To improve biological H_2_ generation, process optimization, genetic modification, metabolic engineering, and reactor design and configuration are investigated. Biofilm is promising, but further research is needed to reduce biofilm formation time, enhance mass transfer through the biofilm matrix, improve light-conversion efficiency and distribution, and develop low-cost, efficient BPBR designs and configurations (Venkatrayalu et al., 2026; Wang et al., 2026).
3. This study reviews current advances and studies on H_2_ production by immobilized photosynthetic bacteria and selection of new high-potential biohydrogen production bacterial strains (Raut et al., 2025c). A long-term immobilized system is advantageous over suspension cultures. Higher H_2_ yields and rates make immobilized systems more appealing and increase cell stability (Zhang et al., 2026).
4. Despite the progress made in generating H_2_, there are still some challenges that need to be overcome for a successful production system. Producing hydrogen in small-scale reactors does not address the challenges of hydraulics, heat, and mass transfer. For example, there have been no reports of a reactor immobilized with a large volume (more than 100 L). To address and overcome the obstacles, it is necessary to implement a reactor of significant size. This will pave the way for future industrial-scale hydrogen production (Mpongwana and Rathilal, 2022; Karimi Alavijeh et al., 2020).
5. In addition, there is a scarcity of literature on running PBRs in real outdoor conditions. Conducted hydrogen production in spring and summer real outdoor conditions, but there is still a need to perform experiments in cold and winter weather conditions. It appears that critical heat transfer modeling is essential for accurately determining the amount of energy needed to create an ideal reaction environment (Sagir and Alipour, 2021).
6. Predicting, analyzing, and optimizing various ways of H_2_ production can also be achieved by artificial intelligence-driven optimization of biohydrogen production, like ANN-GA, RSM, and Python modeling (Raut et al., 2025b). Nanoparticle recycling can reduce the cost of producing newly manufactured nanoparticles (Mpongwana and Rathilal, 2022).

While most studies on H_2_ generation have been conducted at bench scales (i.e., TRL 2–5), there is a lack of adequate academic research and practical investments, making the shift to pilot (TRL 6) and industrial (TRL 7–9) scales a major challenge (Karimi Alavijeh et al., 2020).

## 10 Conclusion

Integrating photocatalytic and photo-fermentative biohydrogen production from LCB with circular bioeconomy and decarbonization goals offers an environmentally sound and scientifically viable approach for future hydrogen economies. This research demonstrates that the use of nanophotocatalysts enhances purple non-sulfur bacteria-driven photofermentation, thereby increasing metabolic efficiency, enzymatic activity, and electron transfer. This enables the efficient utilization of substantial quantities of inexpensive lignocellulosic waste. Despite the positive aspects, obstacles to widespread implementation remain, including inefficient solar-to-hydrogen conversion, high operational costs, photocatalyst deactivation, nanoparticle toxicity, and the inability to scale reactors. According to the TRL evaluation, the majority of biohydrogen production processes involving raw lignocellulosic feedstocks are in the TRL 1–3 range. This highlights the importance of continuous photobioreactor construction, system integration, and the development of visible-light-responsive catalysts. Results from life cycle assessments indicate substantial room for improvement relative to hydrogen processes that rely on fossil fuels, although this depends on factors such as feedstock selection, pretreatment intensity, and energy inputs. Techno-economic analyses indicate that manufacturing costs remain excessively high. Nevertheless, there are ways to scale up, incorporate dark-photo fermentation, recycle catalysts, use inexpensive reactor materials, and reduce the overall cost of the process. Overall, photocatalytic H_2_ production remains in its early stages and is not yet suitable for industrial use. A more efficient and environmentally friendly method for producing H_2_ could be realized with the support of coordinated TEA-LCA frameworks, strengthened academic-industry partnerships, and focused interdisciplinary research.
